# Retreaded tires are an overlooked source of microplastics with distinct additive leaching and ecotoxicity

**DOI:** 10.1038/s43247-026-03566-0

**Published:** 2026-04-28

**Authors:** Hao Liu, Tianchi Cao, Yan Lin, Guoliang Shi, Kaiwei Huang, Zhi Cao, Tong Zhang, Thilo Hofmann, Wei Chen

**Affiliations:** 1 College of Environmental Science and Engineering, Ministry of Education Key Laboratory of Pollution Processes and Environmental Criteria, Tianjin Key Laboratory of Environmental Remediation and Pollution Control, Nankai University, Tianjin, China; 2 State Environmental Protection Key Laboratory of Urban Ambient Air Particulate Matter Pollution Prevention and Control, Nankai University, Tianjin, China; 3 Department of Environmental Geosciences, Centre for Microbiology and Environmental Systems Science, University of Vienna, Vienna, Austria

**Keywords:** Environmental sciences, Environmental social sciences

## Abstract

Retreaded tires constitute a substantial segment of the commercial tire market and are an important source of tire wear particles (TWPs), yet the environmental risks of this major microplastic category remain uninvestigated. Here, we show that although the total additive mass is generally lower in TWPs from retreaded tires, these particles exhibit a markedly greater additive leaching potential, particularly for *p*-phenylenediamines (PPDs). Notably, the highly water-soluble additive *N*-isopropyl-*N’*-phenyl-p-phenylenediamine (IPPD), present at high concentrations in some retreaded-tire TWPs, is especially leachable. Correspondingly, leachates from retreaded-tire TWPs cause greater growth inhibition in *Vibrio fischeri* and *Chlorella vulgaris* than those from new or used tires. Furthermore, our numerical model projections under the Shared Socioeconomic Pathway 2 (SSP2) scenario show that global emissions of retreaded-tire TWPs could increase several hundred-fold by 2060. The substantial and growing risks identified in our study underscore the urgent need for broader investigations into the environmental impacts of these particles.

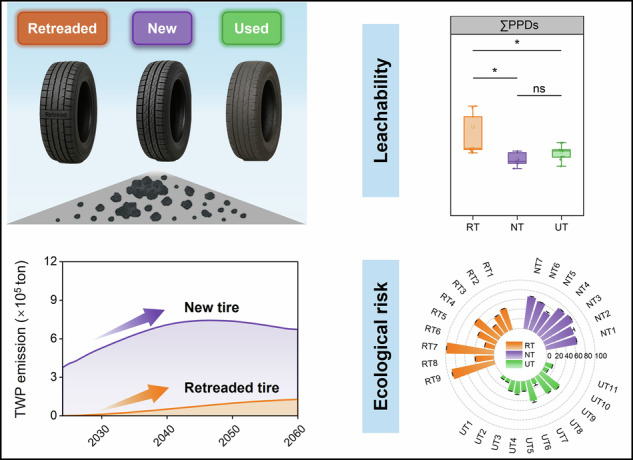

## Abstract (web summary)

Retreaded-tire wear particles show higher leaching of high-risk additives and distinct ecotoxicity than both new and used tires, a risk exacerbated by model projections based on tire sampling and socioeconomic scenarios suggesting global emissions could rise several hundredfold by 2060.

## Introduction

Tire wear particles (TWPs), a major contributor of microplastic pollution in the environment^1–3^, have attracted increasing attention due to their widespread occurrence and adverse ecological impact^4–7^. It is estimated that each year approximately 1.4 million metric tons of TWPs are generated globally, primarily from the mechanical abrasion of tire treads on road surfaces^8,9^. These TWPs are readily transported to soil, aquatic systems, and air via road runoff and atmospheric deposition^10^. TWPs are particulate, polymer-containing materials consisting primarily of natural and synthetic rubber, carbon black, and associated organic and inorganic components^11^. Beyond their physical-mechanical impacts (such as particle ingestion and subsequent impairments to reproduction and survival in freshwater organisms^12,13^), the major concern regarding TWPs lies in the leaching of chemical additives—such as antioxidants, plasticizers, and ultraviolet stabilizers^14^—along with their transformation products and rubber-derived degradation compounds^15–18^. Increasing evidence shows that tire-associated additives and their transformation products exert biological toxicity or adverse physiological effects on diverse aquatic organisms, including fish, zooplankton, and microalgae^19–21^. One high-profile incidence is that *N*-(1,3-dimethylbutyl)-*N’*-phenyl-p-phenylene-diamine quinone (6PPDQ), a transformation product of tire rubber antioxidant *N*-(1,3-dimethylbutyl)-*N’*-phenyl-1,4-benzenediamine (6PPD), causes acute mortality in coho salmon at environmentally relevant low concentrations^22^.

A category of TWPs of particular concern originates from retreaded tires, i.e., tires remanufactured by replacing worn treads with new rubber^23–25^. Retreaded tires represent a substantial segment of the commercial tire market, accounting for approximately 44% of truck tires and up to 90% of aircraft tires in North America^26–28^. Retreads are structurally distinct from both new and worn tires—while the latter two feature an integral tread-to-casing structure (i.e., the tread and casing are manufactured as a single homogeneous unit), retreaded tires may possess a vulcanized interface between the old casing and the new tread^29,30^. Moreover, unlike new tires, which are predominantly produced by a small number of major manufacturers using standardized processes, retreaded tires are mostly produced by small or semi-industrial retreading facilities with variable formulations and practices, conditions that plausibly change additive inventories, leachability, and transformation-product formation (for example, the PPD-family anti-degradants used in heavy-duty casings)^25,27,31,32^. In developing countries, retreaded tires produced with less standardized or even substandard manufacturing procedures are widely in use^23,31,33^. Despite their prevalence and their central role in circular-economy strategies, peer-reviewed environmental and ecotoxicological studies that explicitly treat retreaded tires as a distinct experimental category are essentially absent. This increases the uncertainties in predicting the risks of TWPs. Recent comprehensive reviews of TWPs and guidance documents by the Interstate Technology & Regulatory Council (ITRC) identify products derived from retreads as a clear and urgent knowledge gap^7,34^.

The primary aim of this study is to investigate the distinct environmental risks of TWPs from retreaded tires, including the leaching of toxic additives and transformation products, by systematically comparing their chemical compositions, additive leaching behavior, and leachate ecotoxicity against those from new and used tires, which serve as critical reference controls for benchmarking. We show that although the total additive mass, particularly p-phenylenediamines (PPDs), is generally lower in TWPs from retreaded tires than those from new or aged tires, these particles exhibit a substantially greater additive leaching potential. We further show that the water-soluble additive *N*-isopropyl-*N’*-phenyl-p-phenylenediamine (IPPD), a distinct chemical found in large amounts in some retreaded-tire TWPs, is especially leachable. Accordingly, leachates from retreaded-tire TWPs cause substantially greater growth inhibition in *Vibrio fischeri* and *Chlorella vulgaris* than leachates from either new or used tires. Our model prediction suggests that under the SSP2 scenario, the emission of retreaded tires-derived TWPs will increase by several hundred folds in both developed and developing countries within the next several decades. Thus, the high risks associated with retreaded-tire TWPs underscore the urgent need for broader investigations into the environmental impacts of these particles. Addressing these previously unrecognized trade-offs between material circularity and chemical safety is imperative to ensure that the transition to a circular economy does not inadvertently accelerate the environmental loading of hazardous tire-derived chemicals.

## Results

### Tire wear particles generated from retreaded tires show distinct additive profiles

**Fig. 1:**
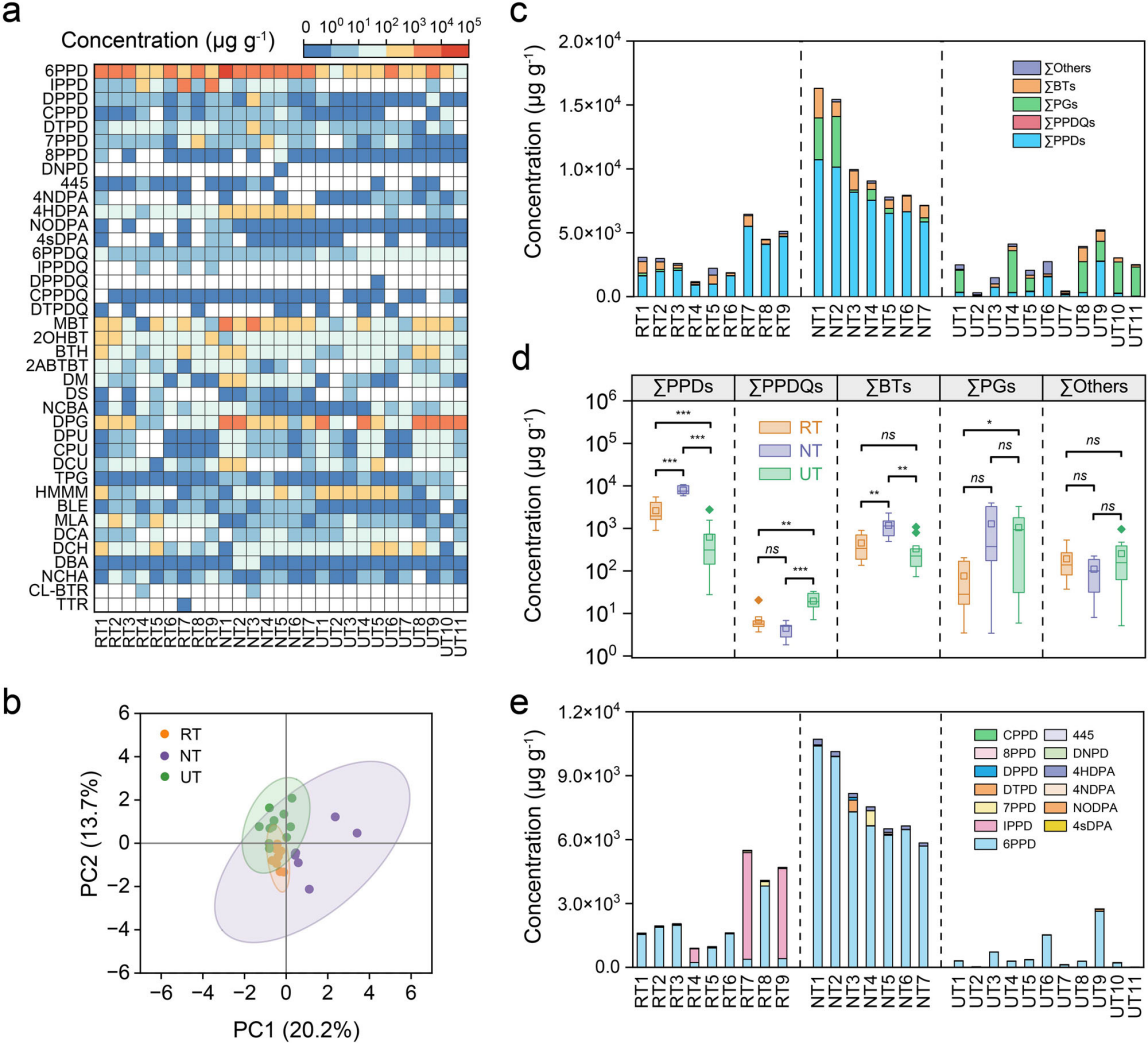
Comparison of chemical compositions of additives in tire wear particles (TWPs) from retreaded with those from new and used tires. **a** Concentrations of chemical additives in TWPs; abbreviations of individual additives are defined in Supplementary Table 1. **b** Principal component analysis (PCA) of chemical compositions in TWPs. **c** Total concentrations (∑) of chemical additives in TWPs. **d** Comparison of concentrations of the five major categories between different types of TWPs. **e** Compositional profiles of *p*-phenylenediamine (PPD) derivatives in TWPs. Shaded ellipses in (**b**) indicate 95% confidence regions of each group. For the box plots in (**d**), center lines indicate medians, boxes indicate the interquartile range (25th–75th percentiles), whiskers extend to 1.5 times the interquartile range (IQR), squares indicate means, and diamond symbols beyond whiskers indicate outliers. The symbols of “_***_”, “_**_”, and “_*_” denote statistically significant differences (*p* < 0.001, *p* < 0.01, and *p* < 0.05, respectively), and “ns” indicates no significant differences.

A screening of a total of 40 chemical additives that are most frequently detected in the environment (Supplementary Table 1) showed that the occurrence and abundance of the additives varied substantially among the TWPs generated from retreaded tires (referred to as “RT” hereafter), new tires (NT), and used tires (UT), with the total mass of the additives generally followed the order of NT > RT > UT. All of the 40 chemical additives were detected across the TWP samples, whereas the detection frequencies of individual compounds and the associated concentrations differed (Fig. 1a and Supplementary Tables 2–4). Between 20 and 27 of the 40 target additives were detected in the 9 RT samples; for the 7 NT samples, 23 to 30 additives were detected, whereas for the 11 UT samples, the number of detected additives ranged from 15 to 26. (The large variability among the UT samples cannot simply be attributed to their service history, as the mass of additives displayed no clear correlations with tread depth reduction or surface O/C ratio, used as a proxy for mileage (Supplementary Fig. 1a, b)^35,36^.) The principal component analysis (PCA) results revealed that the distribution and concentration patterns of the additives in the RT samples were more similar to those in the NT samples, but differed from those in the UT samples (Fig. 1b).

For the RT samples, PPDs accounted for the largest pool of additive mass (the median value of the total concentration of PPDs was 1948.4 μg g^−1^), followed by benzothiazoles (BTs, 287.0 μg g^−1^) and phenyl guanidine (PGs, 28.3 μg g^−1^). The same trend was observed for the NT samples, even though with higher concentrations (7535.7 μg g^−1^ for PPDs, 813.4 μg g^−1^ for BTs, and 375.0 μg g^-1^ for PGs). In contrast, for the UT samples PGs contributed to the largest mass of (936.3 μg g^−1^), followed by PPDs (292.1 μg g^−1^) and BTs (191. 5 μg g^−1^) (Fig. 1c, d). It has been proposed that PPDs (especially 6PPD) are gradually lost from the tire matrix during a tire’s lifespan^37,38^. Consistently, PPD concentrations in the RT samples fell between those in the NT samples and UT samples (Fig. 1d), while the levels of PPDQs, the degradation products of PPDs, showed the reverse trend, reflecting the in-use transformation of PPDs to the oxidized derivatives^39^. The total mass of BTs exhibited a similar trend, that is, the mass of RTs was lower than that of NTs and but higher than that of UTs, whereas the differences in the mass of PGs and the other types of additives among RTs, NTs and UTs were insignificant (Fig. 1d). A detailed examination of individual additives within the PPD pool showed that 6PPD was the predominant PPD additive for most of the TWPs, accounting for as much as 90% of the total PPD mass (Fig. 1e and Supplementary Tables 2–4). However, three RT samples (i.e., RT4, RT7, and RT9) showed a dominant presence (above 80% of total mass) of IPPD instead of 6PPD (Fig. 1e and Supplementary Table 2). Apparently, IPPD was used as an alternative antioxidant by some of the RT manufacturers in place of 6PPD. This is in line with the lack of sufficient quality control in the manufacturing of retreaded tires, as mentioned earlier^31^.

### Tire wear particles from retreaded tires exhibit higher leaching potential

**Fig. 2:**
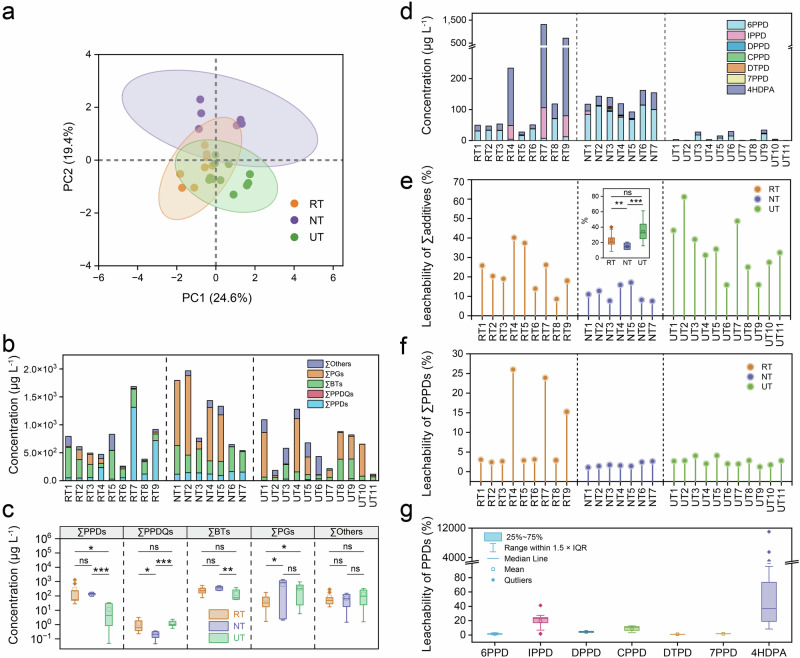
Additive leaching behavior of retreaded-tire TWPs vs TWPs from new and used tires. **a** Principal component analysis (PCA) of chemical compositions in TWP leachates. **b** Total concentrations (∑) of detected chemical additives in TWP leachates. **c** Comparison of concentrations of the five major categories between the leachates from different types of TWPs. **d** Compositional profile of *p*-phenylenediamine (PPD) in TWP leachates. **e** Calculated leachability of total chemical additives in the leachates. **f** Calculated leachability of total PPDs in the leachates. **g** Calculated leachability of individual PPD compounds in the leachates. Note that all leachates were generated using 1 g of TWPs in 1 L of synthetic freshwater. Shaded ellipses in (**a**) indicate 95% confidence regions of each group. For the box plots in (**c**), the inset of **e**, and **g**, center lines indicate medians, boxes indicate the interquartile range (25th–75th percentiles), whiskers extend to 1.5 times the interquartile range (IQR), open squares indicate means, and filled diamond symbols beyond the whiskers indicate outliers. The symbols of “_***_”, “_**_”, and “_*_” denote statistically significant differences (*p* < 0.001, *p* < 0.01, and *p* < 0.05, respectively), and ns indicates no significant differences.

Aqueous leaching experiments revealed that the leachability of the additives from the RT samples differed substantially from that of the NTs and UTs. Among the 40 chemical additives analyzed, only 22 were detected in the leachates (Supplementary Fig. 2). The PCA results revealed that the chemical distribution and concentration patterns in the leachates varied among all three groups of TWPs (Fig. 2a), even though the compositional profiles of the additives in the TWPs were similar between RTs and NTs (Fig. 1b). (Again, for the UTs, no clear correlations were observed between leachable additives and service history; Supplementary Fig. 1c,d.) The relative concentrations of the five groups of additives in the leachates also differed from those in the TWPs. While PPDs contributed predominantly to the total mass of additives in the TWPs (Fig. 1c), the presence of these additives in the leachates was less prominent (Fig. 2b). For the leachates of the 9 RT samples, the concentrations of PPDs were lower than those of BTs for 6 of the samples; the concentrations of both PGs and BTs in the leachates of the NT and UT samples surpassed those of PPDs (Fig. 2b). Additionally, no significant difference in the total concentrations of PPDs was observed between the leachates of RTs and NTs (Fig. 2c), even though in the TWPs the differences were statistically significant (Fig. 1d).

Among the individual PPDs detected in the leachates, 6PPD was the predominant additive for most of the TWPs except for three of the RT samples (Fig. 2d and Supplementary Tables 5–7)—for these three samples, 4HDPA and IPPD were the dominant ones, accounting for nearly 95% of the total PPD mass. The elevated levels of IPPD in the leachates were consistent with its high concentrations in these three TWP samples (Fig. 1e). Note that the heatmap of Spearman’s correlation coefficients among detected chemicals in the TWP leachates revealed a strong positive correlation between 4HDPA and IPPD (Supplementary Fig. 3). This is understandable, as previous research has identified 4HDPA as a major transformation product of IPPD^40^. Overall, the high concentrations of IPPD in these three samples have important implications for the usage of this compound as an alternative to 6PPD.

The calculated leachability data showed that the additives in the RT samples had an overall higher potential of leaching into the aqueous solutions (a mean value of ~20%) than the NT samples (~15%), even though they were less easily leachable than the additives in the UT samples (~30%) (Fig. 2e and Supplementary Fig. 4). It has been shown that microscale channels are formed during the weathering of tires, resulting in increased porosity and reduced crosslink density^5,41,42^. Such structural changes increase water permeability and facilitate additive diffusion and leaching^43^. Thus, the intermediate leaching potential of additives observed for the RT samples likely was due to the mixture of aged carcasses and newly applied treads. Notably, the leaching potential of PPDs was relatively consistent across most samples, ranging between 2% and 4%, regardless of tire category; the only exceptions were the three RT samples containing high concentrations of IPPD (RT4, RT7, and RT9), for which the observed leachability of total PPDs was 26%, 23%, and 15%, respectively (Fig. 2f). This anomalous pattern was primarily driven by the exceptionally high leaching potential of the highly water-soluble IPPD and 4HDPA, the transformation production of IPPD (Fig. 2g).

### IPPD drives the ecotoxicity of tire wear particles from retreaded tires

**Fig. 3:**
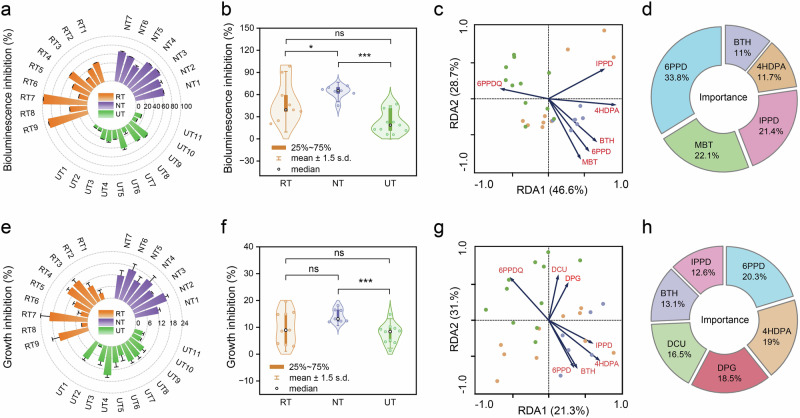
Ecotoxicity evaluation of leachates of retreaded-tire TWPs as compared with those from new and used tires. **a**, **b** Bioluminescence inhibition on *V. fischeri* induced by TWP leachates. **c** Redundancy analysis (RDA) illustrating relationship between the chemical compositions of TWP leachates and the bioluminescence inhibition on *V. fischeri*. **d** Relative importance of individual additives contributing to bioluminescence inhibition on *V. fischeri*, as determined by random forest classification (RFC) models. **e**, **f** TWP leachate induced growth inhibition of *C. vulgaris*, as indicated by reduced cell density. **g** RDA showing the relationship between the chemical composition of TWP leachates and the growth inhibition of *C. vulgaris*. **h** Relative importance of individual additives contributing to growth inhibition of *C. vulgaris*, as determined by RFC models. Error bars in (**a**) and (**e**) indicate standard deviation (s.d.). In the violin plots in (**b**) and (**f**), the outer contours represent the kernel density estimate of the data distribution, boxes indicate the interquartile range (25th–75th percentiles), open circles indicate medians, and capped lines indicate the mean ± 1.5 s.d. The symbols of “_***_” and “_*_” denote statistically significant differences (*p* < 0.001 and *p* < 0.05, respectively), and ns indicates no significant differences.

Considerably larger variances in ecotoxicity were observed among the leachates from the RT samples, as compared with those from the NT and UT samples; in particular, the leachates from the three RTs containing high levels of IPPD exhibited the highest toxicity. *Vibrio fischeri* luminescence inhibition and *C. vulgaris* growth inhibition tests were employed as initial screening assays to assess how the TWPs of retreaded tires may exert environmental impacts differently from those of new and old tires. The variability in bioluminescence inhibition was large among the leachates of the RT samples, a trend also observed for those of UTs but not NTs (Fig. 3a, b). This was consistent with the less standardized processes involved in manufacturing retreaded tires. Among the RT samples, the largest extents of bioluminescence inhibition were exerted by the three samples containing high contents of IPPD, i.e., RT4, RT7, and RT9 (Fig. 3a). Notably, when these outliers were excluded, bioluminescence inhibition exerted by RTs was significantly lower than that by NTs (*p* < 0.001) (Supplementary Fig. 5). Apparently, the high leachability of IPPD and its transformation product, 4HDPA, was the primary cause for the higher toxicity of these three samples. Consistently, redundancy analysis (RDA) identified 4HDPA, IPPD, BTH, 6PPD, MBT, and 6PPDQ as the most important contributors to bioluminescence inhibition (Fig. 3c). Random forest classification (RFC) further indicated that IPPD and 4HDPA made a combined contribution of 33.1% to the overall toxicity, on par with that of 6PPD (33.8%) (Fig. 3d), indicating a strong compound-level association between PPD-type additives and algal growth inhibition. Although the mechanisms controlling the toxicity of IPPD and its transformation product 4HDPA to microalgae have not been directly investigated, studies on structurally related p-phenylenediamine antioxidants, particularly 6PPD and 6PPD-quinone, consistently show that this chemical class induces oxidative stress, disrupts membrane integrity, and interferes with photosynthesis and nutrient metabolism in microalgae^44–46^. Given these shared aromatic structural and redox characteristics among PPD derivatives, IPPD and 4HDPA likely inhibited algal growth through similar mechanisms.

Similar trends were observed in the growth inhibition of *C. vulgaris*. Specifically, variability in the growth inhibition of *C. vulgaris* was much more pronounced among the RT samples than the NT samples (Fig. 3e, f), reflecting greater heterogeneity in additive compositions and leaching potential (Supplementary Tables 5–7), and the three RT samples containing large IPPD mass had larger effects (Fig. 3e, f). Again, RDA and RFC analysis identified 6PPD, IPPD, and 4HDPA as the major contributors to growth inhibition (Fig. 3g), with IPPD and 4HDPA contributing to over 30% of the growth inhibition while 6PPD contributing to an additional 20% (Fig. 3h).

## Discussion

Tire wear particles have been considered a major source of microplastics in the environment. Previous modeling studies estimate that TWPs contribute around 45% of total emitted microplastic loads in both aquatic and terrestrial environments^47,48^. Our study situates retreaded-tire TWPs as a distinct and overlooked subgroup within this major category, possessing environmental risk profiles that diverge from those of both new and used conventional tires. Although TWPs from new tires generally contain higher levels of chemical additives, the greater leachability of additives in TWPs from retreaded tires may result in comparable or even higher environmental risks. The findings highlight a “retreading paradox”: while retreading is traditionally viewed as a sustainable “circular economy” solution, our data reveals critical, previously unrecognized environmental trade-offs.

**Fig. 4:**
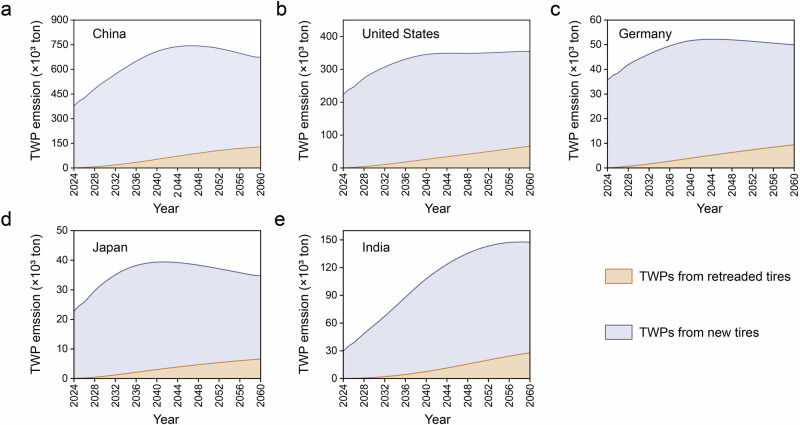
Projected emissions of TWPs from retreaded and new passenger-car tires under the SSP2 scenario for selected countries. **a** China, **b** United States, **c** Germany, **d** Japan, and **e** India. The projections highlight a substantial and steady increase in the emissions of TWPs from retreaded tires in the coming decades.

The United Nations Sustainable Development Goals emphasize the importance of circular economy practices such as remanufacturing and reuse to reduce resource consumption and environmental burdens^49,50^. Retreaded tires are therefore expected to gain substantial market share in the coming decades. We projected emissions of TWPs from retreaded passenger car tires under the SSP2 scenario, assuming a steadily increasing adoption of retreading. The results indicate dynamic growth in retreaded tire-derived TWPs by 2060: a 125-fold increase in China, 200-fold in the US, 180-fold in Germany, 200-fold in Japan, and as much as a 933-fold increase in India (Fig. 4). Thus, the high toxicity of certain additives in TWPs from retreaded tires, along with the variability in additive profiles, leachability, and ecotoxicological effects, underscores the urgent need for close attention to this specific and previously underexplored tire category.

The inherent heterogeneity in retreaded-tire formulations presents a substantial challenge for environmental risk assessment and study reproducibility. Unlike new tires manufactured by major producers under standardized production processes, retreaded tires originate from a comparatively fragmented sector, involving diverse tread compounds and retreading routes (e.g., precure vs. mold-cure), with processing conditions varying across facilities and regions^30,33,51^. This variability likely contributes to the pronounced differences in additive leachability observed among retreaded TWPs. Moreover, the discrepancy between total additive mass and leaching potential suggests that retreading processes may modify the rubber matrix structure (e.g., porosity or bonding characteristics), thereby influencing release dynamics. While our SSP2 modeling projects a substantial expansion of retreaded-tire TWP emissions, the associated environmental impact will depend not only on emission magnitude but also on the chemical profile of future retreads. The elevated toxicity observed in samples with high IPPD content indicates that formulation heterogeneity may generate localized ecological risk hotspots. Although our sampling strategy aimed to capture realistic formulation diversity across multiple sources, broader characterization across global markets will be required to fully constrain this variability. These findings suggest that risk assessment frameworks should account for formulation-dependent leaching behavior, rather than relying solely on bulk additive inventories.

The environmental hazard of retreaded TWPs is a multi-dimensional challenge. The leaching of toxic compounds encompasses not only parent additives (e.g., PPDs) but also their transformation products (e.g., quinones), which are often more toxic. Beyond chemical leaching, the total environmental burden is dictated by the release rate of the particles themselves. Currently, empirical comparisons of emission rates remain scarce; only one peer-reviewed study has directly compared TWP generation from new tire rubber vs. rubber incorporating reclaimed material under controlled laboratory tribological conditions^52^. While these preliminary data suggest that, under typical scenarios, emission rates from retreaded tires are comparable in magnitude with those of conventional tires, it is important to acknowledge that real-world TWP release is governed by the complex interplay of vehicle load, driving behavior, road conditions, and tire formulation. In the absence of systematic comparative field data for retreaded vs. new tires, current emission-factor parameterizations necessarily rely on vehicle-mass scaling approaches. Consequently, future investigations should integrate realistic driving experiments with high-resolution tire characterization to better constrain use-phase emissions of retreaded vs. new and worn tires.

Recent global assessments emphasize the importance of transparency in chemical compositions and international coordination in managing tire additives^15^. Although IPPD has been classified as a Category 1 aquatic acute/chronic toxicant in European regulatory schemes since 1967^53^, and has been largely replaced by 6PPD in some regions^54,55^, our results indicate that this high-risk compound remains present in parts of the global tire inventory. Addressing the risks associated with PPDs and their transformation products will likely require coordinated regulatory and technical approaches. In the near term, downstream stormwater control measures—such as bioretention systems and other green infrastructure—can help intercept roadway-derived chemical loads. Longer-term mitigation may involve upstream measures, including harmonized disclosure of tire and retread formulations, strengthened quality-control standards in retreading practices to reduce formulation-driven variability, and risk-based evaluation or substitution of high-concern antioxidants. Beyond additive-specific concerns, reducing the environmental burden of TWPs will require strategies that limit particle generation and environmental release. Potential approaches include improving material durability and tread design to reduce abrasion-derived emissions, optimizing vehicle operation and maintenance practices, and enhancing interception of tire-derived particles in stormwater and urban runoff systems. Integrating such source-control and interception strategies with additive risk management may provide a more comprehensive framework for mitigating tire-derived microplastic pollution across jurisdictions.

## Conclusions

Retreaded tires constitute a substantial and expanding segment of the commercial tire market and are an important source of TWPs. Despite their prevalence and their central role in the circular economy, peer-reviewed environmental and ecotoxicological studies that explicitly treat retreads as a distinct experimental category are essentially absent. This is a major oversight, as retreads are structurally different from both new and worn tires, and are often produced by smaller facilities with variable formulations and practices, resulting in large uncertainties in the leaching of toxic additives. The findings of this study clearly show that TWPs from retreaded tires exhibit unique additive leaching behaviors (e.g., higher leaching potential of high-risk additives such as IPPD) and ecotoxicological effects that differ from both new and used tires. The significance of these findings is amplified by future projections suggesting a substantial increase in the emission of retreaded tires-derived TWPs worldwide. The distinct and high risks we identified underscore an urgent need to investigate the global environmental impact of this critical emerging microplastic category, and to take into account the chemical dynamics (e.g., leaching and transformation of tire additives, and interactions with environmental media) of retreaded tire products into future regulatory frameworks. Furthermore, achieving a truly sustainable tire lifecycle necessitates standardizing retreading formulations and enhancing additive transparency across the global supply chain. Addressing these previously unrecognized trade-offs between material circularity and chemical safety is imperative to ensure that the transition to a circular economy does not inadvertently accelerate the environmental loading of hazardous tire-derived chemicals.

## Methods

### Sampling of tires

A total of 27 tire samples—including 9 retreaded tires, 7 new tires, and 11 used tires (Supplementary Table 8)—were obtained to prepare the TWP samples. To ensure that our sampling strategy can capture the inherent heterogeneity of the tire retreading industry, our sample pool (*n* = 9) covers a spectrum of manufacturing scales—ranging from small-scale local workshops to medium-scale retreaders—and encompasses both major cold-cure (pre-cure) and mold-cure (hot-cure) processing methods. These samples were strategically sourced from different regions to reflect diverse supply chains of tire casings and retread rubber compounds.

### Materials and chemicals

The detailed information on the materials and chemicals used in this study are provided in Supplementary Note 1. The 40 targeted chemical additives associated with TWPs are classified into five groups based on their chemical structures, including *p*-phenylenediamines (PPDs), quinone derivatives of PPDs (PPDQs), benzothiazoles (BTs), phenyl guanidine (PGs), and other compounds. Selected physicochemical properties of these chemicals are given in Supplementary Table 9.

### Preparation of TWP samples

TWPs were prepared by cryogenic grinding of cleaned tire treads, followed by sieving (<200 µm) and air-drying^56,57^. First, the tire tread surface of each sample was cleaned using moistened laboratory-grade wipes to ensure surface cleanliness prior to processing. The cleaned treads were then manually cut into small fragments using a stainless-steel cutter knife. To produce TWPs, the cut fragments were cryogenically ground using a high-powered fine grinder in the presence of liquid nitrogen to prevent thermal degradation. The resulting ground material was passed through a 200 µm stainless-steel sieve to obtain uniformly sized TWPs. The sieved particles were then air-dried at room temperature for 6 hours to remove residual moisture. The TWP samples prepared with the retreaded tires are referred to as RT1 to RT9, the ones with new tires as NT1 to NT7, and those with used tires as UT1 to UT11. X-ray photoelectron spectroscopy (XPS, PHI 5000 VersaProbe, ULVAC-PHI Inc., Japan) was employed to analyze the surface elemental composition of TWPs, and the O/C atomic ratio was calculated to evaluate the degree of surface oxidation.

### Determination of additive concentrations in TWPs

The concentrations of additives in the TWPs were determined using a solvent extraction method^58^. Each sample was extracted three times with methanol, and the supernatants were collected after centrifugation and combined for analysis. Briefly, approximately 20 mg of each TWP sample was weighed into a glass centrifuge tube with a PTFE-lined cap. Each sample was extracted three times using10 mL of methanol per extraction. The extraction process involved vortexing (~1 min), rotary shaking (~10 min at 60 rpm), and sonication (~20 min). After each extraction, the mixture was centrifuged at 2500 rpm for 20 min, and the resulting supernatants were collected and combined. The combined extracts were evaporated to dryness under a gentle stream of nitrogen and reconstituted in 1 mL of methanol. The final extracts were filtered through 0.22 μm nylon membranes (Anpu Co., China) and diluted 10-fold with methanol for ultra-performance liquid chromatography coupled with a triple quadrupole mass spectrometer (UPLC-MS/MS, Waters Xevo TQ-S, Waters Corporation, USA) analysis. For each TWP sample, three independently prepared replicates were analyzed, and the reported concentrations represent the mean of these replicates.

### Additive leaching experiments

A 100 mg portion of each TWP sample was added to 10 mL of the synthetic freshwater in 20 mL glass bottles^4,59^. The synthetic freshwater was prepared following the United States Environmental Protection Agency guidelines^60^, with the recipe provided in Supplementary Table 10. The samples were placed on a horizontal shaker operated at a constant speed of 125 rpm to simulate dynamic aqueous conditions. Leachates from experimental bottles were collected at exposure time points of 1, 4, 7, and 14 days for subsequent pretreatment and analysis. After collection, all liquid samples were filtered through 0.22 μm glass fiber membranes. The filtered supernatants were then adjusted to 50% (v/v) methanol concentration for UPLC-MS/MS analysis. Leaching experiments for each TWP sample were performed in triplicate. The leaching kinetics indicated that leaching equilibrium was reached in approximately 1 d (Supplementary Fig. 6). The decrease of the concentrations of some additives after reaching the peak was consistent with the transformation of aqueous additives during the course of the experiments^8^. Therefore, the leachates collected at the end of day 1 were used for further analysis of concentration profiles of individual chemicals.

### Chemical analysis and leachability evaluation

Chemical additives were analyzed using UPLC-MS/MS equipped with an electrospray ionization source operated in multiple reaction monitoring mode. Chromatographic separation of target analytes was achieved using an ACQUITY UPLC BEH C18 column (2.1 × 50 mm, 1.7 μm), with a mobile phase consisting of 0.1% formic acid in ultrapure water (A) and methanol (B). The detailed gradient elution program, instrument operating parameters, MRM transitions, collision energies, and retention times for each precursor/product ion pair were optimized and are provided in Supplementary Tables 11 and 12. Quality assurance and quality control (QA/QC) procedures were implemented to ensure analytical reliability. Method blanks were included in each batch, and blank recoveries are summarized in Supplementary Table 13. All reported concentrations were blank-corrected when necessary. Calibration curves were constructed using external standards, with correlation coefficients (*R*^2^) greater than 0.99 for all target compounds. Variations in additive profiles in the TWPs and the leachates were investigated using principal component analysis (PCA). Leachability was defined as the ratio of the concentration of an additive in the leachate to that in the TWP sample from which the leachate was collected.

### Toxicity and ecological risk evaluation

The ecological toxicity of TWP leachates was assessed using bioassays based on *V. fischeri* bioluminescence inhibition (as an indicator of acute toxicity^61^) and *C. vulgaris* growth inhibition (to assess sublethal effects on photosynthetic organisms^62^), as described in Supplementary Notes 2 and 3. Briefly, acute toxicity to *V. fischeri* was determined using the WaterTox^TM^ kit test. The luminescence intensity was measured using a multimode microplate reader (SpectraMax iD5, Molecular Devices, Austria). The inhibition rate (IR%) was calculated based on the changes in luminescence intensity after exposure to the leachate, using Supplementary Eqs. S1 and S2. The ecotoxicity to *C. vulgaris* was evaluated following the standard algal growth inhibition test protocols^63^, with algal density and chlorophyll content monitored for toxicity assessment. Redundancy analysis, together with RFC, was employed to quantify the relative importance of individual chemical compounds in the leachates to the toxicity, as detailed in Supplementary Notes 4 and 5.

### Prediction of TWPs emission under the SSP2 scenario

Future emissions of TWPs from retreaded passenger car tyres were projected using a dynamic material flow analysis (MFA) framework under the SSP2 “middle-of-the-road” scenario, as detailed in Supplementary Note 6. Forty-eight vehicle archetypes, defined by powertrain, size, and lightweighting level, were modeled. Vehicle and tire lifetimes were represented by Weibull distributions (∼15 and ∼3 years, respectively). Retreaded tires were treated as a fraction of retired tyres re-entering the fleet, with the rate assumed to increase linearly to 20% by 2060. Although the framework was applied at the global scale, results are illustrated for five representative countries—China, the United States, Germany, Japan, and India—covering the period 2023–2060.

### Reporting summary

Further information on research design is available in the Nature Portfolio Reporting Summary linked to this article.

## Supplementary information

Reporting Summary

Transparent Peer Review file

Supplementary Information

## Data Availability

The data that support the findings of this study are openly available in Zenodo at 10.5281/zenodo.19633812^64^.

## Code availability

The code used to reproduce the machine-learning analyses reported in this manuscript is openly available in Zenodo at 10.5281/zenodo.19485703^65^.
