# Supplementary material for: Retreaded tires are an overlooked source of microplastics with distinct additive leaching and ecotoxicity
Source: Commun Earth Environ. 2026 Apr 28;7(1):567. doi: 10.1038/s43247-026-03566-0 (PMC13327939; doi:10.1038/s43247-026-03566-0)
Supplement: Supplementary file 1 — Reporting Summary [file 43247_2026_3566_MOESM1_ESM.pdf]

nature portfolio

Corresponding author(s): Thilo Hofmann, Wei Chen

Last updated by author(s): Apr 12, 2026

# Reporting Summary

Nature Portfolio wishes to improve the reproducibility of the work that we publish. This form provides structure for consistency and transparency in reporting. For further information on Nature Portfolio policies, see our Editorial Policies and the Editorial Policy Checklist.

## Statistics

For all statistical analyses, confirm that the following items are present in the figure legend, table legend, main text, or Methods section.

| n/a | Confirmed | |
|---|---|---|
| ☐ | ☒ | The exact sample size (*n*) for each experimental group/condition, given as a discrete number and unit of measurement |
| ☐ | ☒ | A statement on whether measurements were taken from distinct samples or whether the same sample was measured repeatedly |
| ☐ | ☒ | The statistical test(s) used AND whether they are one- or two-sided<br>*Only common tests should be described solely by name; describe more complex techniques in the Methods section.* |
| ☒ | ☐ | A description of all covariates tested |
| ☒ | ☐ | A description of any assumptions or corrections, such as tests of normality and adjustment for multiple comparisons |
| ☐ | ☒ | A full description of the statistical parameters including central tendency (e.g. means) or other basic estimates (e.g. regression coefficient) AND variation (e.g. standard deviation) or associated estimates of uncertainty (e.g. confidence intervals) |
| ☐ | ☒ | For null hypothesis testing, the test statistic (e.g. *F*, *t*, *r*) with confidence intervals, effect sizes, degrees of freedom and *P* value noted<br>*Give P values as exact values whenever suitable.* |
| ☒ | ☐ | For Bayesian analysis, information on the choice of priors and Markov chain Monte Carlo settings |
| ☒ | ☐ | For hierarchical and complex designs, identification of the appropriate level for tests and full reporting of outcomes |
| ☐ | ☒ | Estimates of effect sizes (e.g. Cohen's *d*, Pearson's *r*), indicating how they were calculated |

*Our web collection on statistics for biologists contains articles on many of the points above.*

## Software and code

Policy information about availability of computer code

| | |
|---|---|
| Data collection | OriginPro 2024 |
| Data analysis | Excel2025 , Spss20.0. |

For manuscripts utilizing custom algorithms or software that are central to the research but not yet described in published literature, software must be made available to editors and reviewers. We strongly encourage code deposition in a community repository (e.g. GitHub). See the Nature Portfolio guidelines for submitting code & software for further information.

## Data

Policy information about availability of data

All manuscripts must include a data availability statement. This statement should provide the following information, where applicable:

- Accession codes, unique identifiers, or web links for publicly available datasets
- A description of any restrictions on data availability
- For clinical datasets or third party data, please ensure that the statement adheres to our policy

https://doi.org/10.5281/zenodo.19483497

## Research involving human participants, their data, or biological material

Policy information about studies with human participants or human data. See also policy information about sex, gender (identity/presentation), and sexual orientation and race, ethnicity and racism.

| | |
|---|---|
| Reporting on sex and gender | No human participants, animals or biological material were involved in this study. |
| Reporting on race, ethnicity, or other socially relevant groupings | No human participants, animals or biological material were involved in this study. |
| Population characteristics | No human participants, animals or biological material were involved in this study. |
| Recruitment | No human participants, animals or biological material were involved in this study. |
| Ethics oversight | No human participants, animals or biological material were involved in this study. |

Note that full information on the approval of the study protocol must also be provided in the manuscript.

# Field-specific reporting

Please select the one below that is the best fit for your research. If you are not sure, read the appropriate sections before making your selection.

☐ Life sciences ☐ Behavioural & social sciences ☒ Ecological, evolutionary & environmental sciences

For a reference copy of the document with all sections, see nature.com/documents/nr-reporting-summary-flat.pdf

# Ecological, evolutionary & environmental sciences study design

All studies must disclose on these points even when the disclosure is negative.

| | |
|---|---|
| Study description | This study focuses on tire wear particles (TWPs) derived from retreaded tires, an understudied yet major microplastic source in the commercial tire market. Laboratory experiments reveal that despite lower total additive content, retreaded-tire TWPs display markedly higher leaching potential for p-phenylenediamines (PPDs), especially the highly soluble N-isopropyl-N'-phenyl-p-phenylenediamine (IPPD). Leachates from these particles induce stronger growth inhibition in Vibrio fischeri and Chlorella vulgaris compared to those from new and used tires. Numerical modeling under the SSP2 scenario further predicts that global emissions of retreaded-tire TWPs may increase several hundred-fold by 2060. Collectively, these findings highlight the substantial and rising environmental risks associated with this overlooked microplastic category. |
| Research sample | Tire wear particles (TWPs) from retreaded, new, and used tires were used as research samples. All samples were obtained under simulated laboratory wear conditions for subsequent leaching and toxicity testing. |
| Sampling strategy | To ensure that our sampling strategy can capture the inherent heterogeneity of the tire retreading industry, our sample pool (n = 9) covers a spectrum of manufacturing scales—ranging from small-scale local workshops to medium-scale retreaders—and encompasses both major cold-cure (pre-cure) and mold-cure (hot-cure) processing methods. These samples were strategically sourced from different regions to reflect diverse supply chains of tire casings and retread rubber compounds |
| Data collection | Data collection was performed by the researchers conducting the experiments. Experimental parameters and analytical concentration data were manually recorded in laboratory notebooks and compiled into originPro 2024 software. |
| Timing and spatial scale | All laboratory experiments and instrumental analyses were performed between May 2023 and March 2024.Spatial scale: Retreaded tire samples were collected from different regions in China; new tire samples represented global common tire brands. All laboratory experiments were conducted under controlled indoor conditions. Global emission projections covered the international scale for five representative countries (China, USA, Germany, Japan, India). |
| Data exclusions | No data were excluded from the analyses. |
| Reproducibility | All experiments were conducted in triplicate. Detailed experimental and modeling methods are provided to allow full reproduction of the study. |
| Randomization | Samples were pre-allocated into groups based on their inherent characteristics: retreaded tires, new tires, and used tires. No random allocation was performed, as the groups were defined by tire type prior to the study.To control for potential confounding factors, all samples across groups were subjected to identical experimental conditions, including standardized sample preparation, leaching protocols, and analytical workflows. |
| Blinding | Blinding was not applicable to this study. All experiments were performed under controlled laboratory conditions, and the outcomes measured were objective analytical and numerical data; thus, blinding was not required. |

Did the study involve field work? ☐ Yes ☒ No

# Reporting for specific materials, systems and methods

We require information from authors about some types of materials, experimental systems and methods used in many studies. Here, indicate whether each material, system or method listed is relevant to your study. If you are not sure if a list item applies to your research, read the appropriate section before selecting a response.

## Materials & experimental systems

| n/a | Involved in the study | |
|---|---|---|
| ☒ | ☐ | Antibodies |
| ☒ | ☐ | Eukaryotic cell lines |
| ☒ | ☐ | Palaeontology and archaeology |
| ☒ | ☐ | Animals and other organisms |
| ☒ | ☐ | Clinical data |
| ☒ | ☐ | Dual use research of concern |
| ☐ | ☒ | Plants |

## Methods

| n/a | Involved in the study | |
|---|---|---|
| ☒ | ☐ | ChIP-seq |
| ☒ | ☐ | Flow cytometry |
| ☒ | ☐ | MRI-based neuroimaging |

# Dual use research of concern

Policy information about dual use research of concern

## Hazards

Could the accidental, deliberate or reckless misuse of agents or technologies generated in the work, or the application of information presented in the manuscript, pose a threat to:

| No | Yes | |
|---|---|---|
| ☒ | ☐ | Public health |
| ☒ | ☐ | National security |
| ☒ | ☐ | Crops and/or livestock |
| ☒ | ☐ | Ecosystems |
| ☒ | ☐ | Any other significant area |

## Experiments of concern

Does the work involve any of these experiments of concern:

| No | Yes | |
|---|---|---|
| ☒ | ☐ | Demonstrate how to render a vaccine ineffective |
| ☒ | ☐ | Confer resistance to therapeutically useful antibiotics or antiviral agents |
| ☒ | ☐ | Enhance the virulence of a pathogen or render a nonpathogen virulent |
| ☒ | ☐ | Increase transmissibility of a pathogen |
| ☒ | ☐ | Alter the host range of a pathogen |
| ☒ | ☐ | Enable evasion of diagnostic/detection modalities |
| ☒ | ☐ | Enable the weaponization of a biological agent or toxin |
| ☒ | ☐ | Any other potentially harmful combination of experiments and agents |

## Plants

| | |
|---|---|
| Seed stocks | Chlorella vulgaris was acquired from the Freshwater Algae Culture Collection at the Institute of Hydrobiology (Wuhan, China). |
| Novel plant genotypes | No novel plant genotypes were generated or used in this study |
| Authentication | The Chlorella vulgaris strain used in this study was obtained from the Freshwater Algal Culture Collection at the Institute of Hydrobiology (FACHB collection), where it was previously authenticated by morphological and molecular methods. |
