# Supplementary material for: Retreaded tires are an overlooked source of microplastics with distinct additive leaching and ecotoxicity
Source: Commun Earth Environ. 2026 Apr 28;7(1):567. doi: 10.1038/s43247-026-03566-0 (PMC13327939; doi:10.1038/s43247-026-03566-0)
Supplement: Supplementary file 3 — Supplementary Information [file 43247_2026_3566_MOESM3_ESM.pdf]

Supplementary Information

Retreaded tires are an overlooked source of microplastics with distinct additive leaching and ecotoxicity

Hao Liu[1#], Tianchi Cao[1#], Yan Lin[1], Guoliang Shi[1,2], Kaiwei Huang[1], Zhi Cao[1], Tong Zhang[1], Thilo Hofmann[3]*, and Wei Chen[1]*

[1] College of Environmental Science and Engineering, Ministry of Education Key Laboratory of Pollution Processes and Environmental Criteria, Tianjin Key Laboratory of Environmental Remediation and Pollution Control, Nankai University, Tianjin 300350, China

[2] State Environmental Protection Key Laboratory of Urban Ambient Air Particulate Matter Pollution Prevention and Control, Nankai University, Tianjin 300350, China

[3] Department of Environmental Geosciences, Centre for Microbiology and Environmental Systems Science, University of Vienna, 1090 Vienna, Austria

* Corresponding author: thilo.hofmann@univie.ac.at; chenwei@nankai.edu.cn

[#] These authors contributed equally.

Supplementary Methods

Supplementary Note 1. Materials and chemicals

A total of 40 targeted chemical additives were analyzed in this study. Their full names, abbreviations, CAS registry numbers, and supplier information are listed in Supplementary Tables 1 and 8. These compounds were classified into five categories based on their chemical structures, consistent with previous studies: *p*-phenylenediamines (**PPDs**; including 6PPD, IPPD, DPPD, CPPD, 7PPD, 8PPD, DNPD, DTPD, Naugard 445, 4-HDPA, 4-NDPA, 4s-DPA, and NO-DPA), quinone derivatives of PPDs (**PPDQs**; including 6PPD-Q, IPPD-Q, CPPD-Q, DPPD-Q, and DTPD-Q), benzothiazoles (**BTs**; including MBT, BT, 2-OH-BTH, 2ABTH, DM, DS, and NCBA), phenylguanidine antioxidants (**PGs**; including DPG, TPG, DCU, DPU, CPU, D-DPU, and C-DMU), and **other compounds** (including HMMM, MLA, DCA, DCH, NCHA, DBA, CL-BTR, and TTR). All analytical standards had a purity above 98%. High-performance liquid chromatography (HPLC)-grade methanol (MeOH) was purchased from Merck (Darmstadt, Germany). Formic acid was purchased from CNW (ANPEL Laboratory Technologies Inc., China). Disposable syringe filters with nylon and fiberglass membranes (13 mm diameter and 0.22 μm pore size) were procured from JinTeng (Tianjin, China). The WaterTox^{TM} kit was obtained from EBPI® (Burlington, Canada). *Chlorella vulgaris* was sourced from the Freshwater Algae Culture Collection, Institute of Hydrobiology, Chinese Academy of Sciences (Wuhan, China).

Supplementary Note 2. Assessment of the acute aquatic toxicity of TWP leachates to *Vibrio fischeri*

The acute aquatic toxicity of TWP leachates was assessed using the bioluminescence inhibition assay based on the marine bacterium *Vibrio fischeri*, a well-established bioindicator for rapid toxicity evaluation of environmental contaminants. The assay relies on the principle that toxic substances interfere with the metabolism and/or activity of specific functional proteins in their

bacterial cells, leading to a measurable decrease in their natural bioluminescence. This reduction is quantified to evaluate the toxicity of test samples. Toxicity tests were conducted using was the WaterTox™ kit test, following protocols adapted from previously reported literature[1,2]. Brief, each leachate sample was amended with 2% (w/v) NaCl and tested in triplicate. Blank (2% NaCl) and positive ($ZnSO_4 \cdot 7H_2O$, 9.67 mg L^{-1}) controls were included in parallel with each experimental batch to ensure result validity. Prior to testing, freeze-dried *Vibrio fischeri* powder was rehydrated in 2% NaCl solution. For each measurement, 20 μL of reactivated bacterial suspension and 180 μL of leachate were added to individual wells of a 96-well plate. Plates were incubated at 15 °C for 15 minutes. The luminescence intensity was recorded using a multimode microplate reader (SpectraMax iD5, Molecular Devices, Austria). The inhibition rate (IR%) was calculated using the following equations:

$$C_f = \frac{C_t}{C_0} \quad \text{(S1)}$$

$$IR\% = \frac{S_0 \times C_f - S_t}{S_0 \times C_f} \times 100 \quad \text{(S2)}$$

where C_0 and C_t are the luminescence intensity of *Vibrio fischeri* in 2 wt.% NaCl solution at time = 0 and time = 15 minutes, respectively. C_f is the correction coefficient, S_0 and S_t represent the luminescence intensities of *Vibrio fischeri* exposed to the leachate sample at time = 0 and time = 15 minutes, respectively.

Supplementary Note 3. Assessment of the acute aquatic toxicity of TWP leachates to *C. vulgaris*

The ecotoxicity of TWP leachates was further using the unicellular freshwater green alga, *Chlorella vulgaris*, following standard algal growth inhibition test protocols[3]. Briefly, *Chlorella vulgaris* in BG11 medium within 250 mL conical glass flasks under controlled conditions: 25 °C, 4500 lx in illumination, and light-dark photoperiod in an illumination

incubator (BSG-400, Boxun Medical Biological Instrument, Shanghai, China). Cultures were manually shaken 4–5 times per day to prevent sedimentation. Algal density was measured by a microscope (Olympus Corporation, IX73, Japan), and chlorophyll content was monitored using a UV-Vis spectrophotometry. A calibration curve between chlorophyll absorbance and cell density was established to ensure algae were in the exponential growth phase at the time of exposure (Supplementary Figure 7).

For leachate preparation, TWP samples (10 mg, 100 mg, and 1 g) were added to 100 mL of standard synthetic freshwater in 150 mL glass bottles and shaken at 150 rpm for 24 h on a horizontal shaker. Leachates were then filtered through sterile glass fiber membranes to remove particulates. Algal exposure was conducted in conical flasks containing filtered leachate, fresh medium, and *Chlorella vulgaris* inoculated at an initial density of 1×10^6 cells/mL. All other culture conditions remained consistent with the pre-exposure phase. After four days of incubation, algal cells were harvested by centrifugation for subsequent biochemical analysis.

Chlorophyll (*Chla*) and Chlorophyll b (*Chlb*) content was determined by a colorimetric method. A 4 mL algal suspension was centrifuged at 5000 rpm for 10 min to collect the cells, which were then extracted with 8 mL of ethanol until the cell pellet appeared colorless. Following a 48-h extraction period, the samples were centrifuged again (5000 rpm, 10 min), and the supernatant was analyzed for chlorophyll content. Absorbance at 649 nm (OD_{649}) and 665 nm (OD_{665}) was measured using a UV-Vis spectrophotometer. All measurements were performed in triplicate. Chlorophyll concentration was calculated using the following equations.

$$Chla(\mathrm{mg}\,L^{-1}) = 13.95\mathrm{OD}_{665} - 6.88\mathrm{OD}_{649} \qquad \text{(S3)}$$

$$Chlb(\mathrm{mg}\,L^{-1}) = 24.96\mathrm{OD}_{649} - 7.32\mathrm{OD}_{665} \qquad \text{(S4)}$$

Supplementary Note 4. Principle component analysis and redundancy analysis

Principal component analysis (PCA) was employed to investigate variations in chemical additive profiles among different types of TWPs and their leachates. To identify the specific compounds in TWP leachates that contribute to the growth inhibition of *Vibrio fischeri* and *Chlorella vulgaris*, redundancy analysis (RDA) was performed. Only variables showing statistically significant correlations ($p < 0.05$) were included in the RDA model. All multivariate analyses were conducted using Canoco 5.0.

Supplementary Note 5. Machine learning with random forest classification (RFC)

The relative importances of individual chemical compounds in the leachates on *Vibrio fischeri* luminescence inhibition and *Chlorella vulgaris* growth inhibition were quantified using random forest classification (RFC) implemented in R (version 3.6.3). RFC is a machine learning algorithm capable of capturing non-linear relationships among variables and quantitatively assessing the relative importance of individual predictors in determining environmental outcomes[4]. model development involved several steps. First, input training features were selected based on statistically significant Pearson correlations and redundancy analysis (RDA) of positively associated compounds, and the target variables were the luminescence inhibition rate of *Vibrio fischeri* and the growth inhibition rate of *Chlorella vulgaris*. Second, RFC models were trained separately for *Vibrio fischeri* and *Chlorella vulgaris*, each comprising 81 samples and 6 input features. A total of 500 decision trees were grown (ntree = 500), and two variables were randomly selected at each split (mtry = 2), with hyperparameters optimized to minimize root mean square error (RMSE). Third, a grid search combined with 5-fold cross-validation was used to tune hyperparameters, reduce generalization error, and avoid overfitting. Stratified sampling was applied to maintain the distribution of target variables across folds and mitigate class imbalance. Model performance was evaluated using the F1-score. Finally, each RFC model was trained and tested 50 times using randomly partitioned datasets, and the average F1-score and variable importance rankings were reported

to assess model robustness and identify key predictive features.

Supplementary Note 6. Prediction of TWPs emission under the SSP2 scenario

A global, national-scale modelling framework was developed to project future emissions of TWPs from retreaded passenger car tires under the Shared Socioeconomic Pathway 2 (SSP2), a widely used "middle-of-the-road" scenario describing one possible future development trajectory of human society[5]. While the assessment was conducted at the global scale, five representative countries—China, the United States, Germany, Japan, and India—were selected for illustration, covering the period from 2023 to 2060.

Fleet dynamics were simulated using the dynamic material flow analysis (MFA) approach, which links mobility services (individual motorized transport) to the operation of in-use vehicle stocks and their associated material cycles. The model defines 48 vehicle archetypes, distinguished by six powertrain types (ICEG, ICED, HEV, PHEV, BEV, and FCV), four size categories (microcars, passenger cars, minivans/SUVs, and light trucks), and two lightweighting levels (conventional and lightweighted). Based on available field measurements and emission inventories, mass-based tire wear particle (TWP) generation under realistic driving conditions is primarily governed by vehicle- and use-related factors, including vehicle mass, driving behavior (speed, acceleration, braking), and road surface properties, rather than tire status alone[6,7]. Accordingly, the same TWP emission factor was applied to new (NT), used (UT), and retreaded tires (RT) within the same vehicle archetype, while distinct emission factors were assigned across different vehicle categories (Supplementary Table 14). In the absence of consistent empirical evidence demonstrating systematic differences in use-phase emission rates between new and retreaded tires, this assumption follows current emission-inventory practice and reflects the present state of knowledge.

For each archetype, the total stock of passenger vehicles was calculated from mobility indicators as:

$$Stock = \frac{PKM}{VKM \times OR} \times PoP \quad \text{(S5)}$$

where PKM is passenger-kilometers (average annual distance travelled per person by passenger vehicle); OR is vehicle occupation rate (average number of persons per vehicle per year); VKM is vehicle-kilometers (average annual distance travelled per vehicle); PoP is population. PKM, OR, and VKM values were obtained through existing literature[8,9] and PoP was derived from population projections[10].

A stock-driven dynamic MFA was applied to quantify annual inflows and retirements of passenger vehicles and tires, explicitly including retreaded tires[11,12]. Mathematically, the relationship between new demand and retirements can be expressed as a convolution:

$$Inflow_t = Stock_t - Stock_{t-1} + Outflow_t \quad \text{(S6)}$$

$$Outflow_t = \sum_{t'=t0}^{t'=t-1} Inflow_{t'} \times (1 - S_{t-t'}) \quad \text{(S7)}$$

where $Inflow_t$ or $Inflow_{t'}$ denotes annual demand for passenger vehicles or tires entering the stock at year *t* or *t'*; $Stock_t$ or $Stock_{t-1}$ refers to the in-use stock of passenger vehicles and tires at year t or $t-1$; $Outflow_t$ refers to the number of vehicles or tires retired at year *t*; and $S_{t-t'}$ refers to the probability that the previously vehicle and tires in use reach their end of life after $t-t'$ years.

Vehicle lifetimes followed a Weibull distribution with an average of approximately 15 years[13], while tire lifetimes averaged 3 years[14]. Retreaded tires were modelled as a fraction of retired tires re-entering the fleet in the following year, with the retreading rate assumed to increase linearly to 20% by 2060. Within the dynamic MFA, retreaded tires were treated as a distinct inflow stream, with their in- use stocks, survival, and age structure tracked separately.

TWPs emission was estimated by coupling retreaded tire stocks with archetype-specific emission factors derived from vehicle mass[15,16]. The annual rubber wear rate for each archetype

i in vintage year v, and wear year y was calculated as a function of mileage, tire mass, and emission factor according to:

$$R_{y,v,i} = \frac{EF_i}{T_i \times 10^6} \times \frac{VKM_{y,v,i}}{W_i} \quad \text{(S8)}$$

where $R_{y,v,i}$ is the wear rate for each archetype i in vintage year v and wear year y, EF_i is the emission factor (mg/vehicle/km), T_i is the number of tires per vehicle for archetype i, $VKM_{y,v,i}$ is the annual vehicle mileage of archetype i in year y, and W_i is the average tire mass for archetype i.

Previous studies have reported a close association between TWP emissions and vehicle mass. Early experimental work suggested an approximately linear dependence of TWP emissions on vehicle load[15]. Beddows and Harrison (2021) further identified vehicle mass as a key determinant when comparing TWP emissions across powertrains (for example, BEV versus ICE passenger vehicles)[16], and national-scale emissions inventories likewise use vehicle mass to scale TWP emission factors across vehicle fleets[17]. Accordingly, a mass-based parameterization was used to represent differences in TWP emission factors among vehicle archetypes. Given the limited availability of mass-resolved TWP emission-factor data, vehicle-specific TWP emission factors from an independent real-world measurement study[18] were used as reference values for the scaling and paired with mass data compiled from the literature[8] to assign emission factors to the remaining archetypes. The measurement study reports total TWP emission factors for ICEG passenger cars and HEV passenger cars. These categories were mapped to the corresponding passenger-vehicle archetypes (ICEG/HEV, passenger car, non-lightweight), reflecting the baseline (non-lightweighted) vehicle specification in that study, and used to anchor the mass-scaling applied to the remaining archetypes. This yielded the following form:

$$EF_i = 0.0688V_i - 24.583 \quad \text{(S9)}$$

where V_i is vehicle mass of archtype i. This parameterization was then used to derive archetype-specific EF_i values, as summarized in Supplementary Table 14.

TWPs emissions from retreaded tires were adjusted for by the surviving fraction of tire mass carried over from their previous use, to account for partial wear before retreading. The total annual TWPs emissions in year were calculated as:

$$TWPs_y = \sum_{v=0}^{y} \sum_{i=1}^{48} Stock_{y,v,i} \times T_i \times W_i \times \left(1 - R_{y,v,i}\right)^{(y-v)} \times R_{y,v,i} \qquad \text{(S10)}$$

where $TWPs_y$ is the total TWPs emissions from retreaded tires in year y, $Stock_{y,v,i}$ denotes the number of retreaded tires in use for each archetype i in vintage year v and wear year y, and $\left(1 - R_{y,v,i}\right)^{(y-v)}$ is the cumulative survival of tire mass for each archetype i in vintage year v and wear year y.

Supplementary Table 1. The abbreviations of the 40 selected chemical additives.

Compounds	Full name
6PPD	N-(1,3-Dimethylbutyl)-N′-phenyl-p-phenylenediamine
IPPD	N-isopropyl-N′-phenyl-p-phenylenediamine
DPPD	N, N′-diphenyl-1,4-phenylenediamine
CPPD	N-cyclohexyl-N′-phenyl-p-phenylenediamine
7PPD	4-N-(5-methylhexan-2-yl)-1-N-phenylbenzene-1,4-diamine
8PPD	4-N-octan-2-yl-1-N-phenylbenzene-1,4-diamine
DNPD	N1,N4-di(naphthalen-2-yl)benzene-1,4-diamine
DTPD	N, N′-ditolylp-phenylenediamine
Naugard 445	Bis[4-(2-phenyl-2-propyl) phenyl] amine)
4-HDPA	4-hydroxydiphenylamine
4-NDPA	4-Nitrodiphenylamine
4s DPA	4-Nitrosodiphenylamine
NO-DPA	N, N-Diphenylnitrosamine
6PPD-Q	2,5-dianilinocyclohexa-2,5-diene-1,4-dione
IPPD-Q	2-anilino-5-(propan-2-ylamino)cyclohexa-2,5-diene1,4-dione
CPPD-Q	4-N-(2-methylphenyl)-1-N-(4-methylphenyl)benzene-1,4-diamine
DPPD-Q	2,5-dianilino-p-benzoquinone
DTPD-Q	2,5-bis(2-methylphenylamino)-1,4-benzoquinone
MBT	2-mercaptobenzothiazole
BTH	Benzothiazole
2-OH-BTH	2-hydroxybenzothiazole
2-ABTH	2-benzothiazolamine
DM	2,2'-Dibenzothiazyl disulfide
DS	2-(4-morpholinyldithio)-benzothiazol
NCBA	N-cyclohexyl-1,3-benzothiazol-2-amine
DPG	1,3-diphenylguanidine
TPG	1,2,3-triphenyl-guanidine
DCU	1,3-Dicyclohexylurea
DPU	1,3-diphenylurea
CPU	1-Cyclohexyl-3-phenylurea
D-DPU	1,3-diethyl-1,3-diphenylurea
C-DMU	3-cyclohexyl-1,1-dimethylurea
HMMM	hexa(methoxymethyl)melamine
MLA	Melamine
DCA	N-cyclohexyl-N-methylcyclohexanamine
DCH	Dicyclohexylamine
NCHA	N-cyclohexylformamide

DBA	Dibenzylamine
CL-BTR	5-Chlorobenzotriazole
TTR	4-methyl-1H-benzotriazole

Supplementary Table 2. The concentrations of chemical additives in TWPs from RT samples.

Compounds	RT1 (μg g^{-1})	RT2 (μg g^{-1})	RT3 (μg g^{-1})	RT4 (μg g^{-1})	RT5 (μg g^{-1})	RT6 (μg g^{-1})	RT7 (μg g^{-1})	RT8 (μg g^{-1})	RT9 (μg g^{-1})
6PPD	1549.5	1879.6	1971.0	225.2	909.5	1576.0	381.1	3816.0	410.1
IPPD	1.5	3.6	7.8	648.0	15.5	1.4	5008.6	4.7	4229.2
DPPD	1.8	1.2	3.7	1.3	1.8	0.5	2.0	0.7	3.9
CPPD	0.9	0.3	0.4	4.5	n.d.	0.5	2.4	0.3	2.1
7PPD	6.8	6.6	4.8	1.0	8.1	n.d.	74.9	207.0	5.1
8PPD	1.5	n.d.	n.d.	n.d.	n.d.	n.d.	0.7	0.0	n.d.
DNPD	n.d.	n.d.	n.d.	n.d.	n.d.	n.d.	n.d.	n.d.	n.d.
DTPD	3.5	14.6	15.3	11.8	17.7	7.1	10.0	10.3	25.3
Naugard 445	0.4	0.2	0.7	1.2	0.8	0.4	0.9	n.d.	0.9
4-HDPA	36.0	42.3	44.6	6.0	20.8	36.0	11.0	49.1	11.5
4-NDPA	1.3	n.d.	n.d.	n.d.	n.d.	n.d.	0.4	n.d.	n.d.
4s DPA	0.4	n.d.	n.d.	n.d.	n.d.	n.d.	0.3	n.d.	2.1
NO-DPA	0.4	n.d.	0.0	n.d.	n.d.	n.d.	0.2	n.d.	1.8
6PPD-Q	20.6	6.7	5.2	1.2	5.1	7.1	1.3	3.9	1.7
IPPD-Q	n.d.	n.d.	n.d.	4.4	n.d.	n.d.	2.4	n.d.	3.3
CPPD-Q	n.d.	n.d.	0.4	n.d.	n.d.	n.d.	n.d.	n.d.	n.d.
DPPD-Q	n.d.	n.d.	n.d.	n.d.	n.d.	n.d.	n.d.	n.d.	n.d.
DTPD-Q	n.d.	n.d.	n.d.	n.d.	n.d.	n.d.	n.d.	0.2	n.d.
MBT	236.0	244.3	52.8	0.4	305.2	16.2	334.3	79.9	4.4
BTH	103.3	55.9	35.8	8.1	71.7	45.6	149.0	82.0	28.4
2-OH-BTH	238.0	148.2	74.6	84.7	97.4	74.5	82.8	80.6	103.9
2-ABTH	13.8	16.9	2.8	15.6	6.7	16.6	0.0	17.8	17.3

DM	64.5	4.4	4.4	n.d.	25.0	0.4	33.2	0.6	0.5
DS	n.d.	n.d.	n.d.	n.d.	1.5	n.d.	n.d.	n.d.	n.d.
NCBA	n.d.	25.0	n.d.	26.4	6.9	23.0	5.8	26.0	32.1
DPG	197.7	111.5	137.2	1.7	3.5	n.d.	11.8	14.2	12.7
TPG	0.1	0.1	0.1	0.1	n.d.	n.d.	n.d.	n.d.	n.d.
DCU	5.3	31.2	21.8	48.2	n.d.	16.3	2.7	2.8	14.7
DPU	0.7	6.9	6.0	n.d.	n.d.	0.0	0.1	0.6	0.5
CPU	0.5	19.6	13.9	0.7	0.0	0.2	0.4	0.1	0.4
HMMM	174.8	2.9	3.1	45.4	92.9	n.d.	0.3	4.6	1.1
MLA	39.0	150.7	90.5	8.3	172.7	22.7	35.5	25.3	79.9
DCA	2.8	3.1	1.6	n.d.	12.5	n.d.	n.d.	n.d.	1.2
DCH	107.0	90.8	31.6	12.6	252.7	6.4	74.6	5.4	99.3
NCHA	n.d.	16.8	9.1	11.0	n.d.	7.3	n.d.	3.6	14.9
DBA	n.d.	n.d.	n.d.	n.d.	n.d.	n.d.	n.d.	n.d.	n.d.
CL-BTR	n.d.	n.d.	n.d.	3.2	n.d.	n.d.	n.d.	n.d.	n.d.
TTR	n.d.	n.d.	n.d.	n.d.	n.d.	n.d.	0.9	n.d.	n.d.

Supplementary Table 3. The concentrations of chemical additives in TWPs from NT samples.

Compounds	NT1 (μg g^{-1})	NT2 (μg g^{-1})	NT3 (μg g^{-1})	NT4 (μg g^{-1})	NT5 (μg g^{-1})	NT6 (μg g^{-1})	NT7 (μg g^{-1})
6PPD	10391.68	9890.26	7295.95	6638.22	6200.49	6467.69	5694.96
IPPD	44.00	8.85	10.00	16.00	10.00	2.30	1.75
DPPD	1.70	0.15	124.00	1.04	3.25	0.46	0.46
CPPD	5.60	2.50	1.50	6.50	11.55	1.95	0.75
7PPD	1.97	7.80	3.45	696.50	33.80	2.30	1.85
8PPD	n.d.	n.d.	n.d.	n.d.	75.27	n.d.	n.d.
DNPD	n.d.	n.d.	n.d.	n.d.	0.79	n.d.	n.d.
DTPD	1.32	1.37	542.50	3.56	8.40	1.14	1.46
Naugard 445	n.d.	n.d.	5.89	0.10	n.d.	n.d.	n.d.
4-HDPA	262.74	219.64	182.67	173.14	163.89	163.18	138.27
4-NDPA	0.74	n.d.	n.d.	n.d.	0.72	n.d.	n.d.
4s DPA	1.79	n.d.	0.20	0.26	0.17	0.41	0.48
NO-DPA	1.73	0.15	0.24	0.33	0.35	0.47	0.54
6PPD-Q	1.83	4.37	2.81	5.04	6.47	4.83	4.85
IPPD-Q	n.d.	n.d.	n.d.	n.d.	n.d.	n.d.	n.d.
CPPD-Q	n.d.	0.11	n.d.	0.12	0.15	n.d.	0.38
DPPD-Q	n.d.	n.d.	n.d.	n.d.	n.d.	n.d.	n.d.
DTPD-Q	1.83	4.37	2.81	5.04	6.47	4.83	4.85
MBT	1169.61	559.56	1078.99	263.27	345.96	831.60	672.56
BTH	195.53	106.82	89.49	68.73	66.34	87.80	58.89
2-OH-BTH	7.51	10.23	26.66	23.11	57.72	28.38	31.67
2-ABTH	25.37	10.80	4.14	n.d.	1.07	n.d.	2.92

DM	188.82	109.74	61.87	25.14	20.73	23.21	14.10
DS	n.d.	0.36	1.26	0.23	0.22	2.24	0.05
NCBA	35.38	15.85	3.98	n.d.	n.d.	n.d.	n.d.
DPG	2864.45	3563.34	171.02	802.94	348.88	3.29	307.38
TPG	4.83	5.51	0.20	1.00	0.86	n.d.	0.94
DCU	169.06	184.65	n.d.	n.d.	n.d.	n.d.	5.55
DPU	25.47	38.72	1.85	8.28	3.30	0.14	2.37
CPU	205.48	169.80	1.69	33.28	21.90	n.d.	4.72
HMMM	n.d.	0.05	1.12	1.64	7.37	1.10	0.00
MLA	n.d.	5.82	9.39	10.82	27.71	2.92	1.39
DCA	0.17	36.65	23.41	49.97	56.49	30.44	23.94
DCH	5.02	n.d.	n.d.	4.40	7.04	n.d.	n.d.
NCHA	n.d.	72.86	11.20	n.d.	n.d.	n.d.	n.d.
DBA	n.d.	n.d.	1.13	n.d.	n.d.	n.d.	n.d.
CL-BTR	n.d.	n.d.	n.d.	n.d.	n.d.	n.d.	n.d.
TTR	n.d.	0.05	1.12	1.64	7.37	1.10	n.d.

Supplementary Table 4. The concentrations of chemical additives in TWPs from UT samples.

Compounds	UT1 (μg g^{-1})	UT2 (μg g^{-1})	UT3 (μg g^{-1})	UT4 (μg g^{-1})	UT5 (μg g^{-1})	UT6 (μg g^{-1})	UT7 (μg g^{-1})	UT8 (μg g^{-1})	UT9 (μg g^{-1})	UT10 (μg g^{-1})	UT11 (μg g^{-1})
6PPD	294.5	25.5	713.5	289.5	353.0	1508.9	116.5	290.5	2630.6	204.0	12.9
IPPD	n.d.	n.d.	n.d.	n.d.	n.d.	n.d.	n.d.	n.d.	2.2	n.d.	n.d.
DPPD	1.4	n.d.	n.d.	n.d.	n.d.	n.d.	n.d.	n.d.	18.9	n.d.	n.d.
CPPD	0.3	n.d.	0.5	0.9	0.6	n.d.	n.d.	n.d.	0.8	14.4	n.d.
7PPD	n.d.	n.d.	2.6	n.d.	6.1	11.0	7.5	n.d.	0.8	0.4	n.d.
8PPD	n.d.	n.d.	n.d.	n.d.	n.d.	n.d.	n.d.	n.d.	n.d.	n.d.	n.d.
DNPD	n.d.	n.d.	n.d.	n.d.	n.d.	n.d.	n.d.	n.d.	n.d.	n.d.	n.d.
DTPD	10.3	2.2	2.2	1.3	4.3	2.0	1.2	0.6	88.8	2.8	n.d.
Naugard 445	n.d.	n.d.	n.d.	n.d.	0.1	n.d.	n.d.	n.d.	0.3	n.d.	n.d.
4-HDPA	n.d.	n.d.	n.d.	n.d.	n.d.	2.5	n.d.	n.d.	2.6	3.0	n.d.
4-NDPA	0.6	0.4	0.5	0.4	1.9	1.4	2.5	0.8	1.2	0.7	1.5
4s DPA	n.d.	n.d.	n.d.	n.d.	n.d.	0.3	n.d.	n.d.	0.3	n.d.	n.d.
NO-DPA	n.d.	n.d.	0.1	n.d.	n.d.	0.3	n.d.	n.d.	0.2	n.d.	n.d.
6PPD-Q	18.2	7.1	8.1	18.1	32.1	30.7	17.6	17.4	19.4	24.7	13.9
IPPD-Q	n.d.	n.d.	n.d.	n.d.	n.d.	n.d.	n.d.	n.d.	n.d.	n.d.	n.d.
CPPD-Q	0.3	n.d.	n.d.	0.4	0.7	n.d.	0.3	0.2	n.d.	4.9	0.3
DPPD-Q	n.d.	n.d.	n.d.	n.d.	0.2	n.d.	n.d.	n.d.	n.d.	n.d.	n.d.
DTPD-Q	0.6	n.d.	n.d.	0.4	n.d.	0.2	n.d.	n.d.	n.d.	n.d.	n.d.
MBT	14.2	5.1	87.0	126.2	51.4	62.3	2.5	491.4	353.8	121.7	5.6
BTH	6.2	1.9	24.1	39.0	15.7	18.9	4.5	142.7	104.3	34.4	4.4
2-OH-BTH	44.1	62.1	80.2	65.3	87.6	47.5	71.9	70.0	55.1	28.7	75.6
2-ABTH	n.d.	n.d.	n.d.	n.d.	15.9	5.7	21.7	6.9	7.2	21.2	28.3

DM	0.5	n.d.	6.0	1.8	0.6	0.6	n.d.	24.0	20.9	4.1	0.5
DS	n.d.	n.d.	2.9	n.d.	n.d.	n.d.	n.d.	n.d.	n.d.	n.d.	n.d.
NCBA	n.d.	n.d.	n.d.	n.d.	20.3	1.4	27.5	9.3	9.3	28.5	37.3
DPG	1593.4	15.6	n.d.	3102.4	755.6	11.0	13.3	1968.2	1465.3	1606.0	113.1
TPG	0.9	n.d.	n.d.	3.1	0.9	n.d.	n.d.	6.1	2.0	7.5	n.d.
DCU	34.1	11.3	5.9	50.1	114.3	n.d.	74.8	n.d.	n.d.	78.9	10.1
DPU	42.5	1.8	n.d.	56.0	14.8	0.1	0.3	20.0	11.4	28.2	1.7
CPU	53.3	2.5	n.d.	62.2	50.8	0.1	1.1	6.3	3.7	61.3	2.3
HMMM	264.9	115.2	371.8	145.5	239.2	544.4	1.1	0.6	11.9	2.1	10.8
MLA	1.3	3.8	10.4	1.0	3.9	3.6	1.6	n.d.	1.8	n.d.	1.1
DCA	7.9	3.4	10.6	13.1	25.2	85.4	n.d.	n.d.	2.5	n.d.	2.1
DCH	67.6	34.0	81.1	18.5	116.0	325.6	56.5	108.8	72.4	0.6	18.9
NCHA	2.7	n.d.	n.d.	1.1	2.2	n.d.	2.5	n.d.	n.d.	2.4	n.d.
DBA	n.d.	n.d.	n.d.	n.d.	n.d.	n.d.	n.d.	n.d.	n.d.	n.d.	n.d.
CL-BTR	n.d.	n.d.	n.d.	n.d.	n.d.	n.d.	n.d.	n.d.	n.d.	n.d.	n.d.
TTR	n.d.	n.d.	n.d.	n.d.	n.d.	n.d.	n.d.	n.d.	n.d.	n.d.	n.d.

Supplementary Table 5. The concentrations of chemical additives in the leachates of TWPs from RT samples.

**Compounds**	**RT1 (μg L^{-1})**	**RT2 (μg L^{-1})**	**RT3 (μg L^{-1})**	**RT4 (μg L^{-1})**	**RT5 (μg L^{-1})**	**RT6 (μg L^{-1})**	**RT7 (μg L^{-1})**	**RT8 (μg L^{-1})**	**RT9 (μg L^{-1})**
6PPD	32.44	32.45	29.98	5.45	16.23	37.47	8.14	68.08	11.60
IPPD	0.48	1.14	1.53	78.59	4.70	0.99	196.88	1.80	134.67
DPPD	n.d.	n.d.	n.d.	n.d.	n.d.	n.d.	n.d.	n.d.	n.d.
CPPD	n.d.	n.d.	n.d.	n.d.	n.d.	n.d.	n.d.	n.d.	n.d.
7PPD	n.d.	n.d.	n.d.	n.d.	n.d.	n.d.	n.d.	n.d.	n.d.
8PPD	n.d.	n.d.	n.d.	n.d.	n.d.	n.d.	n.d.	n.d.	n.d.
DNPD	n.d.	n.d.	n.d.	n.d.	n.d.	n.d.	n.d.	n.d.	n.d.
DTPD	n.d.	n.d.	n.d.	n.d.	n.d.	n.d.	n.d.	n.d.	n.d.
Naugard 445	n.d.	n.d.	n.d.	n.d.	n.d.	n.d.	n.d.	n.d.	n.d.
4-HDPA	18.60	13.08	20.23	185.78	8.99	12.46	1206.34	46.96	635.49
4-NDPA	n.d.	n.d.	n.d.	n.d.	n.d.	n.d.	n.d.	n.d.	n.d.
4s DPA	n.d.	n.d.	n.d.	n.d.	n.d.	n.d.	0.10	n.d.	0.10
NO-DPA	n.d.	n.d.	n.d.	n.d.	n.d.	n.d.	n.d.	n.d.	n.d.
6PPD-Q	1.87	0.57	0.57	0.13	0.54	0.88	0.12	0.50	0.19
IPPD-Q	n.d.	n.d.	n.d.	1.55	n.d.	n.d.	1.26	n.d.	2.13
CPPD-Q	n.d.	n.d.	n.d.	n.d.	n.d.	n.d.	n.d.	n.d.	n.d.
DPPD-Q	n.d.	n.d.	n.d.	n.d.	n.d.	n.d.	n.d.	n.d.	n.d.
DTPD-Q	n.d.	n.d.	n.d.	n.d.	n.d.	n.d.	n.d.	n.d.	n.d.
MBT	228.69	115.17	79.20	6.46	197.27	48.42	155.14	93.35	8.99
BTH	97.95	94.41	67.62	n.d.	81.75	40.16	100.41	69.64	26.95
2-OH-BTH	207.65	112.94	84.46	64.55	84.43	66.66	61.61	53.90	75.45
2-ABTH	n.d.	n.d.	n.d.	n.d.	n.d.	n.d.	n.d.	n.d.	n.d.

DM	10.02	2.99	2.49	n.d.	7.03	1.21	8.82	2.76	n.d.
DS	n.d.	n.d.	n.d.	n.d.	0.53	n.d.	n.d.	n.d.	n.d.
NCBA	n.d.	n.d.	n.d.	n.d.	n.d.	n.d.	n.d.	n.d.	n.d.
DPG	6.95	92.82	113.83	0.99	1.60	n.d.	9.84	11.33	9.59
TPG	n.d.	n.d.	n.d.	n.d.	n.d.	n.d.	n.d.	n.d.	0.11
DCU	10.06	46.91	34.77	80.90	n.d.	30.84	n.d.	5.19	24.00
DPU	0.31	9.63	7.18	0.07	0.24	0.14	0.18	0.68	0.65
CPU	0.84	25.32	21.30	1.04	n.d.	0.41	0.97	0.41	0.63
HMMM	113.18	2.83	1.90	35.79	52.94	n.d.	0.27	4.23	1.16
MLA	17.08	32.12	84.42	59.86	90.07	24.41	101.03	11.34	21.81
DCA	2.60	2.05	0.83	0.71	8.64	n.d.	0.27	1.91	0.70
DCH	62.15	50.62	23.19	25.85	159.66	17.34	33.28	19.77	46.75
NCHA	2.12	21.11	15.69	31.71	3.26	17.56	0.96	18.28	14.72
DBA	n.d.	n.d.	n.d.	n.d.	n.d.	n.d.	n.d.	n.d.	n.d.
CL-BTR	n.d.	n.d.	n.d.	n.d.	n.d.	n.d.	n.d.	n.d.	n.d.
TTR	n.d.	n.d.	n.d.	n.d.	n.d.	n.d.	n.d.	n.d.	n.d.

Supplementary Table 6. The concentrations of chemical additives in the leachates of TWPs from NT samples.

Compounds	NT1 ($\mu g\ L^{-1}$)	NT2 ($\mu g\ L^{-1}$)	NT3 ($\mu g\ L^{-1}$)	NT4 ($\mu g\ L^{-1}$)	NT5 ($\mu g\ L^{-1}$)	NT6 ($\mu g\ L^{-1}$)	NT7 ($\mu g\ L^{-1}$)
6PPD	113.47	96.64	91.47	76.06	68.86	104.10	94.24
IPPD	18.06	3.81	4.39	7.70	4.67	1.20	0.81
DPPD	n.d.	n.d.	6.22	n.d.	n.d.	n.d.	n.d.
CPPD	0.32	n.d.	n.d.	1.00	1.43	n.d.	n.d.
7PPD	n.d.	n.d.	n.d.	2.25	n.d.	n.d.	n.d.
8PPD	n.d.	n.d.	n.d.	n.d.	n.d.	n.d.	n.d.
DNPD	n.d.	n.d.	n.d.	n.d.	n.d.	n.d.	n.d.
DTPD	n.d.	n.d.	5.76	n.d.	n.d.	n.d.	n.d.
Naugard 445	n.d.	n.d.	n.d.	n.d.	n.d.	n.d.	n.d.
4-HDPA	22.10	29.95	30.71	36.74	21.24	47.18	54.01
4-NDPA	n.d.	n.d.	n.d.	n.d.	n.d.	n.d.	n.d.
4s DPA	0.27	n.d.	0.11	0.09		0.08	0.14
NO-DPA	n.d.	n.d.	n.d.	n.d.	n.d.	n.d.	n.d.
6PPD-Q	0.20	0.45	0.40	0.62	0.70	0.60	0.32
IPPD-Q	n.d.	n.d.	n.d.	n.d.	n.d.	n.d.	n.d.
CPPD-Q	n.d.	n.d.	n.d.	n.d.	n.d.	n.d.	n.d.
DPPD-Q	n.d.	n.d.	n.d.	n.d.	n.d.	n.d.	n.d.
DTPD-Q	n.d.	n.d.	n.d.	n.d.	n.d.	n.d.	n.d.
MBT	357.87	210.31	280.12	142.04	136.64	291.58	237.49
BTH	139.02	82.61	113.12	69.25	60.94	112.38	94.01
2-OH-BTH	8.99	10.98	26.54	21.35	48.55	24.00	26.54
2-ABTH	n.d.	n.d.	n.d.	n.d.	n.d.	n.d.	n.d.

DM	10.73	7.83	12.95	6.05	4.90	9.53	6.43
DS	n.d.	n.d.	n.d.	n.d.	n.d.	n.d.	n.d.
NCBA	n.d.	n.d.	n.d.	n.d.	n.d.	n.d.	n.d.
DPG	565.16	822.05	52.08	282.64	129.34	0.61	39.08
TPG	0.25	0.39	n.d.	0.10	0.18	n.d.	0.03
DCU	281.96	305.41	5.48	266.33	473.19	1.86	n.d.
DPU	22.22	35.46	1.57	7.19	2.81	n.d.	1.59
CPU	270.16	228.88	2.94	60.44	40.40	n.d.	n.d.
HMMM	0.55	40.67	30.57	46.02	49.87	11.83	3.73
MLA	n.d.	n.d.	n.d.	n.d.	7.42	n.d.	n.d.
DCA	n.d.	4.12	6.05	9.21	20.75	1.79	0.56
DCH	0.84	19.64	14.77	35.12	39.81	20.21	12.47
NCHA	7.76	5.16	4.76	8.36	14.30	n.d.	n.d.
DBA	n.d.	20.65	5.61	n.d.	n.d.	n.d.	n.d.
CL-BTR	n.d.	n.d.	n.d.	n.d.	n.d.	n.d.	n.d.
TTR	n.d.	n.d.	n.d.	n.d.	n.d.	n.d.	n.d.

Supplementary Table 7. The concentrations of chemical additives in the leachates of TWPs from UT samples.

Compounds	UT1 (μg L^{-1})	UT2 (μg L^{-1})	UT3 (μg L^{-1})	UT4 (μg L^{-1})	UT5 (μg L^{-1})	UT6 (μg L^{-1})	UT7 (μg L^{-1})	UT8 (μg L^{-1})	UT9 (μg L^{-1})	UT10 (μg L^{-1})	UT11 (μg L^{-1})
6PPD	3.27	n.d.	19.81	3.34	9.31	15.42	0.98	3.03	22.91	1.55	n.d.
IPPD	n.d.	n.d.	n.d.	n.d.	n.d.	n.d.	n.d.	n.d.	0.78	n.d.	n.d.
DPPD	n.d.	n.d.	n.d.	n.d.	n.d.	n.d.	n.d.	n.d.	0.71	n.d.	n.d.
CPPD	n.d.	n.d.	n.d.	n.d.	n.d.	n.d.	n.d.	n.d.	n.d.	n.d.	n.d.
7PPD	n.d.	n.d.	n.d.	n.d.	n.d.	n.d.	n.d.	n.d.	n.d.	n.d.	n.d.
8PPD	n.d.	n.d.	n.d.	n.d.	n.d.	n.d.	n.d.	n.d.	n.d.	n.d.	n.d.
DNPD	n.d.	n.d.	n.d.	n.d.	n.d.	n.d.	n.d.	n.d.	n.d.	n.d.	n.d.
DTPD	n.d.	n.d.	n.d.	n.d.	n.d.	n.d.	n.d.	n.d.	1.43	n.d.	n.d.
Naugard 445	n.d.	n.d.	n.d.	n.d.	n.d.	n.d.	n.d.	n.d.	n.d.	n.d.	n.d.
4-HDPA	n.d.	n.d.	9.72	n.d.	n.d.	14.46	n.d.	n.d.	10.40	3.12	n.d.
4-NDPA	n.d.	n.d.	n.d.	n.d.	n.d.	n.d.	n.d.	n.d.	n.d.	n.d.	n.d.
4s DPA	n.d.	n.d.	n.d.	n.d.	n.d.	n.d.	n.d.	n.d.	n.d.	n.d.	n.d.
NO-DPA	n.d.	n.d.	n.d.	n.d.	n.d.	n.d.	n.d.	n.d.	n.d.	n.d.	n.d.
6PPD-Q	1.78	0.67	0.82	1.55	3.15	1.69	1.21	0.89	1.35	1.35	1.26
IPPD-Q	n.d.	n.d.	n.d.	n.d.	n.d.	n.d.	n.d.	n.d.	n.d.	n.d.	n.d.
CPPD-Q	n.d.	n.d.	n.d.	n.d.	n.d.	n.d.	n.d.	n.d.	n.d.	n.d.	n.d.
DPPD-Q	n.d.	n.d.	n.d.	n.d.	n.d.	n.d.	n.d.	n.d.	n.d.	n.d.	n.d.
DTPD-Q	n.d.	n.d.	n.d.	n.d.	n.d.	n.d.	n.d.	n.d.	n.d.	n.d.	n.d.
MBT	16.76	7.80	116.82	72.49	34.35	28.85	4.57	234.32	227.52	52.98	6.58
BTH	n.d.	n.d.	51.32	28.31	49.02	54.82	n.d.	87.90	82.77	n.d.	n.d.
2-OH-BTH	40.17	47.27	69.21	47.57	54.62	38.31	52.50	54.96	36.15	20.84	60.17
2-ABTH	n.d.	n.d.	n.d.	n.d.	n.d.	n.d.	n.d.	n.d.	n.d.	n.d.	n.d.

DM	n.d.	n.d.	3.30	1.39	0.83	0.55	n.d.	5.58	6.10	0.95	n.d.
DS	n.d.	n.d.	0.01	n.d.	n.d.	n.d.	n.d.	n.d.	n.d.	n.d.	n.d.
NCBA	n.d.	n.d.	n.d.	n.d.	n.d.	n.d.	n.d.	n.d.	n.d.	n.d.	n.d.
DPG	577.83	8.00	n.d.	684.21	168.92	3.29	5.77	435.74	380.27	340.08	3.43
TPG	n.d.	0.53	0.13	n.d.	n.d.	0.39	0.14	0.24	n.d.	n.d.	n.d.
DCU	69.57	18.68	10.14	88.08	97.54	1.98	120.80	5.25	4.60	117.74	17.14
DPU	49.62	1.35	0.06	53.35	16.04	0.15	0.41	19.17	13.08	30.37	0.57
CPU	95.09	3.74	n.d.	107.77	40.32	0.31	1.61	9.00	5.15	83.28	2.31
HMMM	173.81	74.52	215.10	129.12	186.36	249.20	1.18	0.46	9.44	1.34	8.76
MLA	n.d.	n.d.	11.65	n.d.	n.d.	n.d.	n.d.	n.d.	n.d.	n.d.	n.d.
DCA	8.03	2.60	7.83	15.13	14.21	16.62	n.d.	n.d.	0.56	n.d.	1.90
DCH	46.69	19.77	56.99	22.98	52.68	60.64	26.57	18.78	15.67	0.23	13.50
NCHA	6.72	3.10	3.31	8.13	11.43	4.94	4.28	2.73	3.03	3.51	2.61
DBA	n.d.	n.d.	n.d.	n.d.	n.d.	n.d.	n.d.	n.d.	n.d.	n.d.	n.d.
CL-BTR	n.d.	n.d.	n.d.	n.d.	n.d.	n.d.	n.d.	n.d.	n.d.	n.d.	n.d.
TTR	n.d.	n.d.	n.d.	n.d.	n.d.	n.d.	n.d.	n.d.	n.d.	n.d.	n.d.

Supplementary Table 8. Selected information of the tires used for the generation of TWPs.

**No.**	**Brand**	**Sidewall markings**	**Suitable for the vehicle models**	**Manufacturing date**
RT1	FENGSHENG	1100/R20	Truck	2024
RT2	CHIWEITE	1100/R20	Truck	2024
RT3	CHIWEITE	1100/R20	Truck	2024
RT4	BAOYUANXING	825/R20	Truck	2024
RT5	BAISHENGWEI	1100/R20	Truck	2024
RT6	HONGYUN	650/R16	Van	2024
RT7	HUAQIANG	900/R20	Truck	2024
RT8	LIBEN	700/R16	Van	2024
RT9	CHIWEITE	825/R20	Truck	2024
NT1	SIOECHOY	235/45/R18	Sedan	2024
NT2	MICHELIN	255/50/R20	Sport utility vehicle	2024
NT3	GITI	205/55/R16	Sedan	2024
NT4	GITI	225/55/R17	Sedan	2024
NT5	LINGLONG	175/R14	Van	2024
NT6	LINGLONG	1100/R20	Truck	2024
NT7	LINGLONG	825/R20	Truck	2024
UT1	GITI	225/50/R17	Sedan	2018
UT2	LINGLONG	175/R14	Van	2018
UT3	LINGLONG	1100/R20	Truck	2018
UT4	HANKOOK	205/55/R16	Sedan	2018
UT5	CHAOYANG	215/60/R16	Sedan	2018
UT6	CINTURATO	245/45/R18	Sedan	2018
UT7	ANTTNA	205/55/R16	Sedan	2018
UT8	BRIDGESTONE	195/55/R16	Sedan	2018
UT9	GITI	215/55/R18	Sedan	2018
UT10	MICHELIN	225/65/R17	Sport utility vehicle	2018
UT11	WARRIO	185/65/R14	Sedan	2018

Supplementary Table 9. Selected physicochemical properties of the 40 selected additives.

Compounds	Brand	CAS	Molecular formula	Molecular weight (g mol^{-1})	Log K_{OW}	Water solubility (mg L^{-1})
6PPD	J&K	793-24-8	C18H24N2	268.4	4.68	2.841
IPPD	AccuStandard	101-72-4	C15H18N2	226.3	3.28	50.32
DPPD	AccuStandard	74-31-7	C18H16N2	260.3	4.04	1.277
CPPD	Fluorochem	101-87-1	C18H22N2	266.4	4.64	2.083
7PPD	TRC	3081-01-4	C19H26N2	282.4	5.17	0.5915
8PPD	TRC	15233-47-3	C20H28N2	296.4	5.74	0.1627
DNPD	J&K	93-46-9	C26H20N2	360.4	6.39	0.00146
DTPD	TRC	27417-40-9	C20H20N2	288.4	5.13	0.5914
Naugard 445	Mreda	10081-67-1	C30H31N	405.6	9.82	1.16E-05
4-HDPA	Macklin	122-37-2	C12H11NO	185.22	2.46	202.8
4-NDPA	Aladdin	836-30-6	C12H10N2O2	214.22	-1.16	3.52E+05
4s DPA	Mreda	156-10-5	C12H10N2O	198.22	3.16	89.17
NO-DPA	Macklin	86-30-6	C12H10N2O	198.22	3.16	94.85
6PPD-Q	HPC	2754428-18-5	C18H22N2O2	298.38	4	51
IPPD-Q	HPC	68054-73-9	C15H16N2O2	256.3	2.58	2020
CPPD-Q	HPC	68054-78-4	C18H20N2O2	296.4	3.94	94.79
DPPD-Q	HPC	3421-08-7	C18H14N2O2	290.3	3.5	15
DTPD-Q	HPC	1005173-46-5	C20H18N2O2	318.4	4.6	1.2
MBT	Sigma Aldrich	149-30-4	C7H5NS2	167.3	2.86	554.2
BTH	Sigma Aldrich	95-16-9	C7H5NS	135.19	2.17	1684

2-OH-BTH	Sigma Aldrich	934-34-9	C7H5NOS	151.19	2.35	2354
2-ABTH	TRC	136-95-8	C7H6N2S	150.2	2	1477
DM	Tmstandard	120-78-5	C14H8N2S4	332.49	4.66	0.06439
DS	Macklin	95-32-9	C11H12N2OS3	284.42	1.59	657.6
NCBA	Macklin	28291-75-0	C13H16N2S	232.34	4.82	2.266
DPG	Bidepharm	102-06-7	C13H13N3	211.26	2.89	129.1
TPG	Macklin	101-01-9	C19H17N3	287.36	5.04	0.7183
DCU	Macklin	2387-23-7	C13H24N2O	224.34	3.92	46.76
DPU	Macklin	102-07-8	C13H12N2O	212.25	2.97	103.7
CPU	Sigma Aldrich	886-59-9	C13H18N2O	218.29	3.44	151.4
D-DPU	Macklin	85-98-3	C17H20N2O	268.35	4.20	4.791
C-DMU	Sigma Aldrich	31468-12-9	C9H18N2O	170.25	0.65	1.71E+05
HMMM	Macklin	3089-11-0	C15H30N6O6	390.44	1.61	149.3
MLA	Macklin	108–78–1	C3H6N6	126.12	-1.37	1E+006
DCA	Sigma Aldrich	7560-83-0	C13H25N	195.34	4.58	319.4
DCH	Macklin	101-83-7	C12H23N	181.32	4.37	103.1
NCHA	Macklin	766-93-8	C7H13NO	127.18	1.12	1.033E+004
DBA	Macklin	103-49-1	C14H15N	197.28	3.24	2414
CL-BTR	TCI	94-97-3	C6H4ClN3	153.57	1.81	2077
TTR	Sigma Aldrich	136-85-6	C7H7N3	133.15	1.71	3069
6PPD-Q-d5	HPC	2750119-14-1	C18H17D5N2O2	303.41	/	/
BTH-d4	TRC	164423-51-3	C7HD4NS	139.211	/	/
Atrazine-d5	HPC	163165-75-1	C8H9D5ClN5	220.71	/	/

Supplementary Table 10. Formulation of synthetic freshwater.

	Reagent Added (mg L^{-1})				Approximate Final Water Quality		
	$NaHCO_3$	$CaSO_4 \cdot 2H_2O$	$MgSO_4$	KCl	pH[1]	Hardness[2]	Alkalinity[2]
Moderately Hard	96.0	60.0	60.0	4.0	7.4-7.8	80-100	57-64

[1] Approximate equilibrium pH after 24 h of aeration.

[2] Expressed as mg ($CaCO_3$) L^{-1}.

Supplementary Table 11. Detailed information on gradient elution.

Time (min)	%A[1]	%B[2]
0	90	10
3	90	10
5	30	70
5.1	0	100
7	0	100
7.1	90	10
9	90	10

[1] 0.1% formic acid in ultrapure water (v/v).

[2] Methanol.

Supplementary Table 12. UPLC-MS/MS operating parameters and precursor/product ion transitions for the 40 selected additives.

Chemicals	Retention time (min)	Precursor ions (m/z)	Production ions (m/z)	Dwell (s)	Cone (V)	Collision (V)	Internal standard
6PPD	3.64	269.05	184.1	0.003	40	25	6PPD-Q-d5
			212		40	25	
IPPD	2.83	227.03	92.6	0.003	40	33	6PPD-Q-d5
			107		40	33	
DPPD	3.82	261.00	183.8	0.003	40	20	6PPD-Q-d5
			167.9		40	20	
CPPD	3.07	267.03	92.7	0.003	40	24	6PPD-Q-d5
			183.9		40	24	
7PPD	3.23	283.37	80.1	0.003	64	26	6PPD-Q-d5
			107.08		64	26	
8PPD	3.62	297.39	92.98	0.003	62	32	6PPD-Q-d5
			107.03		62	32	
DNPD	3.96	360.4	217.19	0.003	40	32	6PPD-Q-d5
			232.93		40	32	
DTPD	4.08	289	197.9	0.003	20	24	6PPD-Q-d5
			180.8		20	24	
Naugard 445	4.41	406.26	91.02	0.003	60	45	6PPD-Q-d5
			196		60	35	
4-HDPA	3.06	186	80.21	0.003	72	34	6PPD-Q-d5
			109.1		72	34	
4-NDPA	3.65	215	167.1	0.003	30	15	6PPD-Q-d5

			198.2		30	10	
4s DPA	3.19	199	128	0.003	20	35	6PPD-Q-d5
			181		20	20	
NO-DPA	3.23	199	76.93	0.003	15	20	6PPD-Q-d5
			168.9		15	20	
6PPD-Q	4.91	299.2	241.1	0.003	40	30	6PPD-Q-d5
			99.97		40	20	
IPPD-Q	3.23	257.09	107	0.003	40	33	6PPD-Q-d5
			92.6		40	33	
CPPD-Q	3.71	297.31	97.93	0.003	28	32	6PPD-Q-d5
			186.99		28	32	
DPPD-Q	3.82	291.27	77.05	0.003	20	44	6PPD-Q-d5
			143.87		20	44	
DTPD-Q	3.64	319.3	117.95	0.003	40	34	6PPD-Q-d5
			128.19		40	34	
MBT	2.96	168	123.8	0.003	40	22	BT-d4
			134.8		40	22	
BTH	2.98	120.03	95	0.003	34	22	BT-d4
			109		34	22	
2-OH-BTH	2.85	151.97	80.03	0.003	78	20	BT-d4
			119		78	20	
2ABTH	3.06	151.04	64.88	0.003	16	26	BT-d4
			123.87		16	26	
DM	2.45	333.28	167	0.003	20	20	BT-d4
			198		20	20	

DS	2.7	285.05	118	0.003	15	15	BT-d4
			200		15	10	
NCBA	3.15	233.1	55.05	0.003	60	30	BT-d4
			151		60	25	
DPG	2.41	212.1	93.99	0.003	98	10	Atrazine-d5
			65.06		98	10	
TPG	2.82	288.2	91.95	0.003	10	45	Atrazine-d5
			194.7		10	45	
DCU	3.37	225.34	82.9	0.003	40	20	Atrazine-d5
			100		40	20	
DPU	3.11	213.03	77.03	0.003	10	30	Atrazine-d5
			94		10	30	
CPU	3.26	219.2	94.07	0.003	5	18	Atrazine-d5
			77.15		5	18	
D-DPU	3.09	269.13	120.03	0.003	10	25	Atrazine-d5
			148.04		10	15	
C-DMU	3.32	171.21	72.03	0.003	15	18	Atrazine-d5
			89.07		15	12	
HMMM	3.03	391	177	0.003	40	10	Atrazine-d5
			207		40	10	
MLA	2.38	127	85	0.003	15	15	Atrazine-d5
			67.9		15	21	
DCA	2.51	196.12	55.11	0.003	88	32	Atrazine-d5
			41.07		88	32	
DCH	4.2	182.3	55.09	0.003	5	26	Atrazine-d5

			83.11		5	18	
NCHA	3.45	128	55	0.003	15	15	Atrazine-d5
			83		15	15	
DBA	3.55	198.13	83	0.003	15	10	Atrazine-d5
			106.06		15	15	
CL-BTR	3.12	153.98	90	0.003	15	15	Atrazine-d5
			98		15	15	
TTR	3.41	134.07	79.03	0.003	10	15	Atrazine-d5
			77.1		10	15	

Supplementary Table 13. Method blank recoveries and limits of quantification (LOQs) for the chemical additives detected in TWPs.

Compounds	Recovery (%)	LOQ[1] (μg g^{-1})
6PPD	85.19	2.3
IPPD	83.16	1.01
DPPD	80.2	0.004
CPPD	88.23	0.226
7PPD	87.54	0.518
8PPD	86.78	0.59
DNPD	91.18	0.27
DTPD	67.17	0.475
Naugard 445	81.01	0.06
4-HDPA	55.86	0.52
4-NDPA	78.66	0.31
4s DPA	79.61	0.15
NO-DPA	72.24	0.033
6PPD-Q	91.29	0.15
IPPD-Q	84.73	0.12
CPPD-Q	92.41	0.053
DPPD-Q	87.19	0.058
DTPD-Q	85.55	0.016
MBT	67.23	1.3
BTH	71.59	2.69
2-OH-BTH	89.29	2.14
2-ABTH	89.60	2.58
DM	147.78	0.42
DS	130.25	0.0083
NCBA	56.14	0.26
DPG	96.94	1.15
TPG	83.08	0.031
DCU	94.82	2.49
DPU	91.12	0.087
CPU	88.62	0.061
HMMM	91.48	0.17
MLA	65.74	1.1
DCA	55.93	0.64
DCH	54.16	0.011
NCHA	38.98	0.83
DBA	46.15	0.42

CL-BTR	96.94	0.031
TTR	101.88	0.73

[1]LOQs were calculated based on a signal-to-noise (S/N) ratio of 10.

Supplementary Table 14. TWP emission factors for different vehicle categories.

Archetype	TWP emission factor (mg/vehicle/km)
ICEG, microcar, lightweight	16.53
ICEG, microcar, non-lightweight	25.64
ICEG, passenger car, lightweight	52.95
ICEG, passenger car, non-lightweight	53.00
ICEG, minivan/SUVs, lightweight	72.07
ICEG, minivan/SUVs, non-lightweight	93.27
ICEG, light truck, lightweight	97.19
ICEG, light truck, non-lightweight	115.63
ICED, microcar, lightweight	17.22
ICED, microcar, non-lightweight	26.50
ICED, passenger car, lightweight	54.06
ICED, passenger car, non-lightweight	65.96
ICED, minivan/SUVs, lightweight	73.33
ICED, minivan/SUVs, non-lightweight	94.83
ICED, light truck, lightweight	99.12
ICED, light truck, non-lightweight	117.90
BEV, microcar, lightweight	30.89
BEV, microcar, non-lightweight	47.23
BEV, passenger car, lightweight	74.35
BEV, passenger car, non-lightweight	93.27
BEV, minivan/SUVs, lightweight	92.13
BEV, minivan/SUVs, non-lightweight	125.90
BEV, light truck, lightweight	126.23
BEV, light truck, non-lightweight	155.12
HEV, microcar, lightweight	19.12
HEV, microcar, non-lightweight	30.75
HEV, passenger car, lightweight	57.36
HEV, passenger car, non-lightweight	72.00
HEV, minivan/SUVs, lightweight	75.44
HEV, minivan/SUVs, non-lightweight	101.74
HEV, light truck, lightweight	102.28
HEV, light truck, non-lightweight	128.02
PHEV, microcar, lightweight	19.54
PHEV, microcar, non-lightweight	33.09
PHEV, passenger car, lightweight	58.25
PHEV, passenger car, non-lightweight	74.97

PHEV, minivan/SUVs, lightweight	75.08
PHEV, minivan/SUVs, non-lightweight	105.26
PHEV, light truck, lightweight	102.28
PHEV, light truck, non-lightweight	128.02
FCV, microcar, lightweight	18.26
FCV, microcar, non-lightweight	31.64
FCV, passenger car, lightweight	56.60
FCV, passenger car, non-lightweight	73.32
FCV, minivan/SUVs, lightweight	76.04
FCV, minivan/SUVs, non-lightweight	107.18
FCV, light truck, lightweight	101.46
FCV, light truck, non-lightweight	127.47

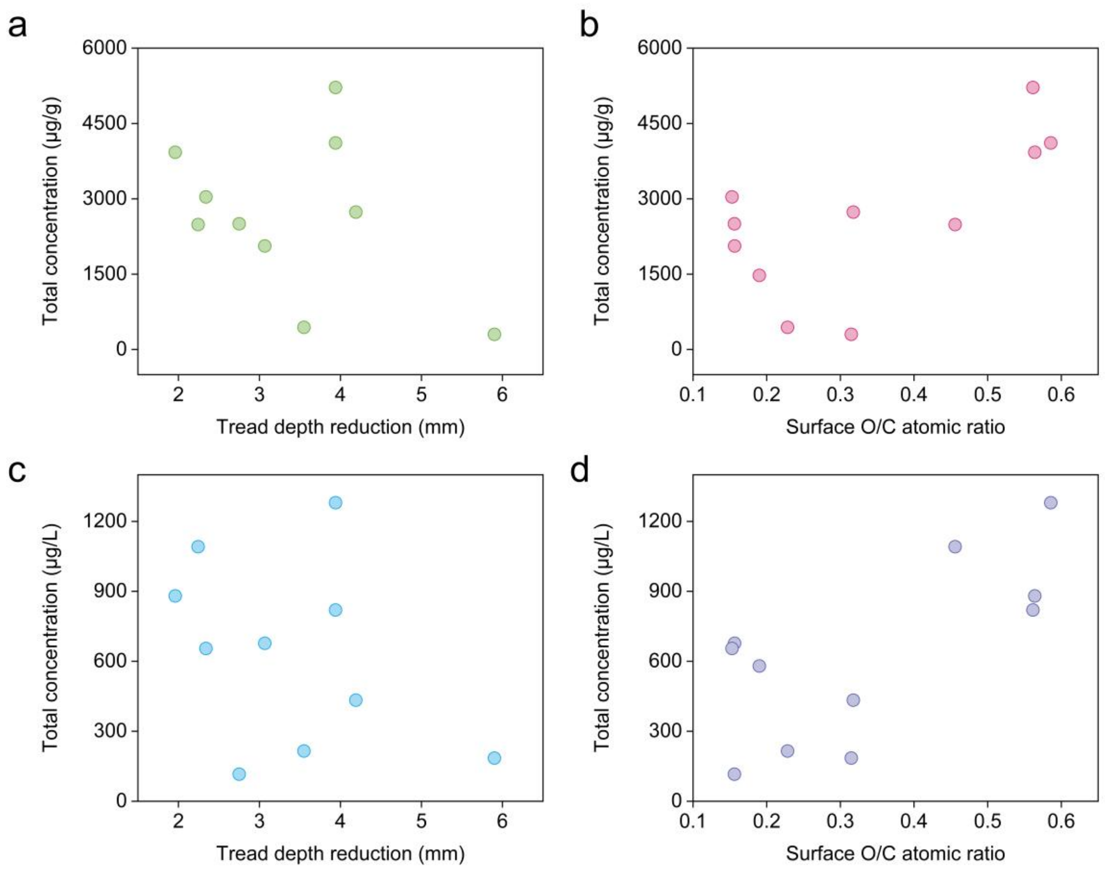

Supplementary Figure 1. Plot of total concentrations of chemical additives in the TWPs from used tires (**a** and **b**) and concentrations in TWP leachates (**c** and **d**) against the tread depth reduction (i.e., thickness of tread lost during service) and degree of surface oxidation (expressed as the O/C atomic ratio measured by XPS).

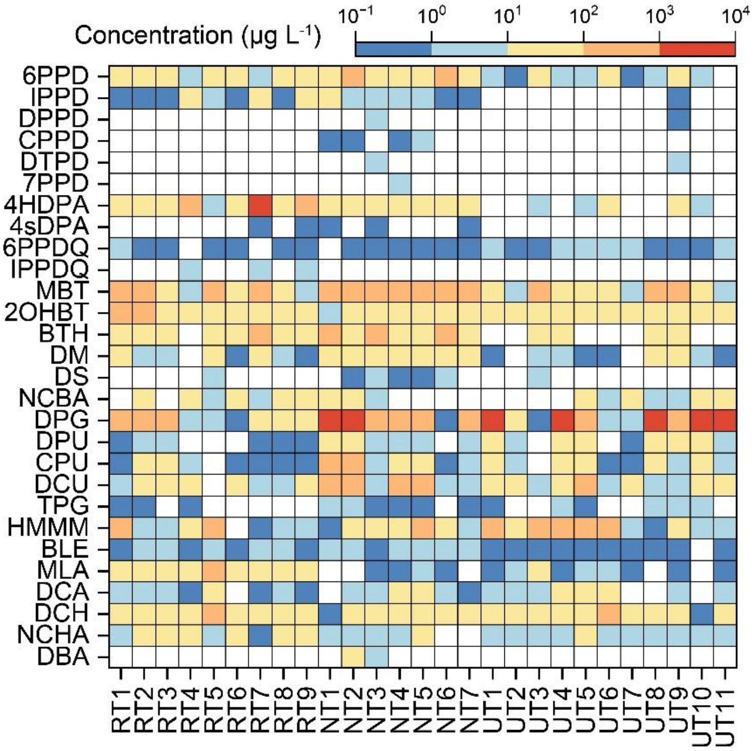

Supplementary Figure 2. Concentrations of individual chemical additives in TWP leachates.

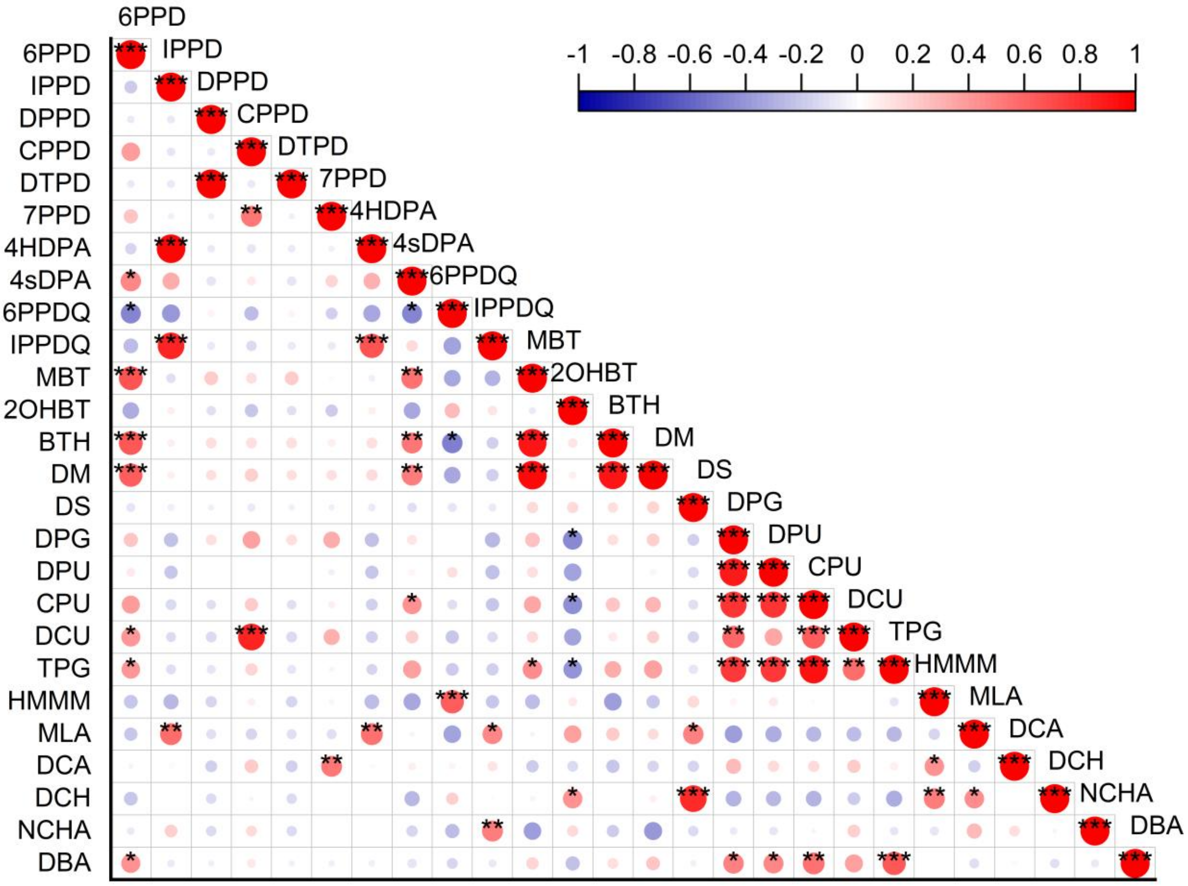

Supplementary Figure 3. Heatmap of Spearman's correlation coefficients among detected chemical additives in TWP leachates. The symbols of "***", "**", and "*" denote statistically significant differences ($p < 0.001$, $p < 0.01$, and $p < 0.05$, respectively).

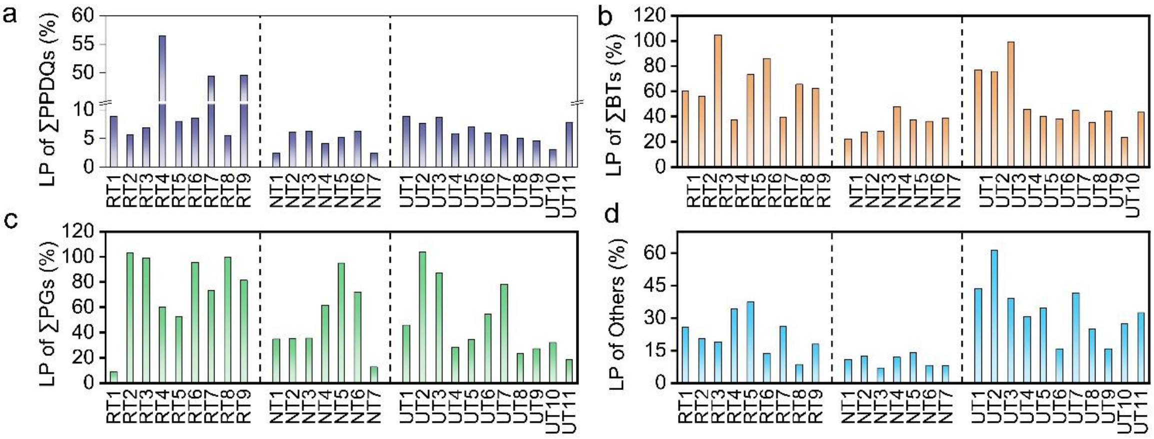

Supplementary Figure 4. Leachability of different categories of chemical additives: **a** ∑PPDQs, **b** ∑BTs, **c** ∑PGs, and **d** Others. All leachates were generated using 1 g of TWPs in 1 L of synthetic freshwater. The acronym LP stands for leaching potential.

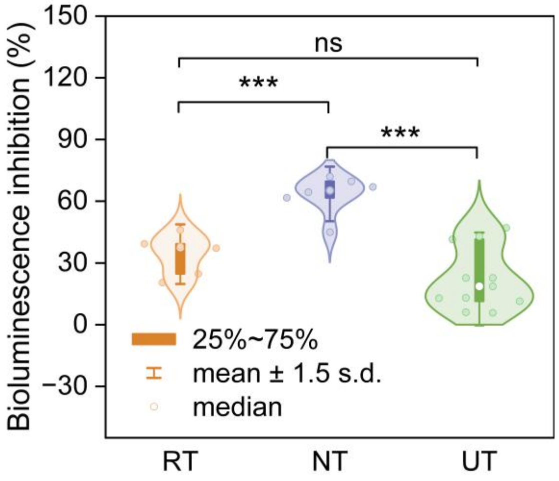

Supplementary Figure 5. Bioluminescence inhibition on *Vibrio fischeri* induced by TWP leachates (RT4, RT7, and RT9 are excluded). The symbols of "***" donate statistically significant differences ($p < 0.001$). The expression of "ns" represents no significant difference. For the violin plots, the outer contours represent the kernel density estimate of the data distribution, boxes indicate the interquartile range (25th–75th percentiles), open circles indicate medians, and capped lines indicate the mean ± 1.5 s.d. All leachates were generated using 1 g of TWPs in 1 L of synthetic freshwater.

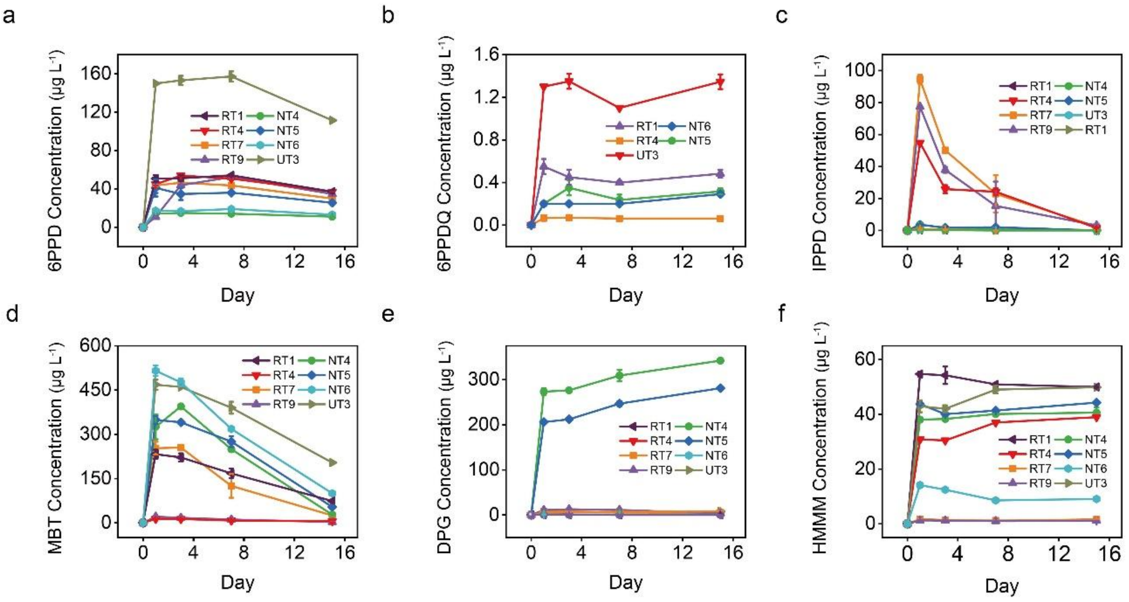

Supplementary Figure 6. Leaching kinetics of representative chemical additives **a** 6PPD, **b** 6PPDQ, **c** IPPD, **d** MBT, **e** DPG, and **f** HMMM in representative TWP samples. Error bars represent standard deviation (s.d.).

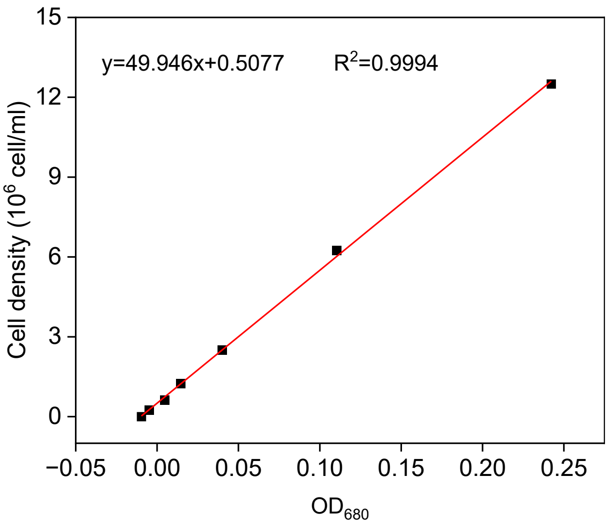

Supplementary Figure 7. Correlation between the density of *Chlorella vulgaris* and OD_{680}.

Supplementary References

1. Wang, W., et al. Toxicity of substituted p-phenylenediamine antioxidants and their derived novel quinones on aquatic bacterium: Acute effects and mechanistic insights. *J. Hazard. Mater.* **469**, 133900 (2024).

2. Amariei, G., et al. First eco-toxicological evidence of ivabradine effect on the marine bacterium Vibrio fischeri: A chiral view. *Sci. Total Environ.* **838**, 156617 (2022).

3. Li, L. A., et al. Marine heatwaves exacerbate the toxic effects of tire particle leachate on microalgae. *Environ. Sci. Technol.* **59**, 177-187 (2025).

4. Du, T., Shi, G., Liu, F., Zhang, T. & Chen, W. Sulfidation of Ag and ZnO nanomaterials significantly affects protein corona composition: Implications for human exposure to environmentally aged nanomaterials. *Environ. Sci. Technol.* **53**, 14296-14307 (2019).

5. O'Neill, B. C., et al. The roads ahead: Narratives for shared socioeconomic pathways describing world futures in the 21st century. *Glob. Environ. Chang.-Hum. Policy Dimens.* **42**, 169-180 (2017).

6. Grigoratos, T. & Martini, G. Non-exhaust traffic related emissions: Brake and tyre wear PM. Report EUR 26648 (2014).

7. Fussell, J. C., et al. A review of road traffic-derived non-exhaust particles: emissions, physicochemical characteristics, health risks, and mitigation measures. *Environ. Sci. Technol.* **56**, 6813-6835 (2022).

8. Pauliuk, S., et al. Global scenarios of resource and emission savings from material efficiency in residential buildings and cars. *Nat. Commun.* **12**, 5097 (2021).

9. Pauliuk, S., et al. Linking service provision to material cycles: A new framework for studying the resource efficiency-climate change (RECC) nexus. *J. Ind. Ecol.* **25**, 274-287 (2021).

10. Samir, K. C. & Lutz, W. The human core of the shared socioeconomic pathways: Population scenarios by age, sex and level of education for all countries to 2100. Glob. *Environ. Change-Hum. Policy Dimens.* **42**, 181-192 (2017).

11. Cao, Z., et al. Resourcing the fairytale country with wind power: A dynamic material flow analysis. *Environ. Sci. Technol.* **53**, 11313-11322 (2019).

12. Liu, G., Bangs, C. E. & Müller, D. B. Stock dynamics and emission pathways of the global aluminium cycle. *Nat. Clim. Chang.* **3**, 338-340 (2013).

13. Modaresi, R., Pauliuk, S., Lovik, A. N. & Müller, D. B. Global carbon benefits of material substitution in passenger cars until 2050 and the impact on the steel and aluminum industries. *Environ. Sci. Technol.* **48**, 10776-10784 (2014).

14. Cui, D., et al. Scenario analysis of waste tires from China's vehicles future. *J. Clean. Prod.* **478**, 143940 (2024).

15. Wang, C., Huang, H. B., Chen, X. & Liu, J. P. The influence of the contact features on the tyre wear in steady-state conditions. *Proc. Inst. Mech. Eng. D J. Automob. Eng.* **231**, 1326-1339 (2017).

16. Beddows, D. C. S. & Harrison, R. M. PM10 and PM2.5 emission factors for non-exhaust particles from road vehicles: Dependence upon vehicle mass and implications for battery electric vehicles. *Atmos. Environ.* **244**, 117886 (2021).

17. Polukarova, M., Hjort, M. & Gustafsson, M. Comprehensive approach to national tire wear emissions: Challenges and implications. *Sci. Total Environ.* **924**, 171391 (2024).

18. Liu, Y., et al. Impact of vehicle type, tyre feature and driving behaviour on tyre wear under real-world driving conditions. *Sci. Total Environ.* **842**, 156950 (2022).
