# Supplementary material for: Retreaded tires are an overlooked source of microplastics with distinct additive leaching and ecotoxicity
Source: Commun Earth Environ. 2026 Apr 28;7(1):567. doi: 10.1038/s43247-026-03566-0 (PMC13327939; doi:10.1038/s43247-026-03566-0)
Supplement: Supplementary file 2 — Transparent Peer Review file [file 43247_2026_3566_MOESM2_ESM.pdf]

Peer Review File

Retreaded tires are an overlooked source of microplastics with distinct additive leaching and ecotoxicity

Corresponding Author: Professor Thilo Hofmann

This file contains all editorial decision letters in order by version, followed by all author rebuttals in order by version.

Version 0:

Decision Letter:

** Please ensure you delete the link to your author home page in this e-mail if you wish to forward it to your coauthors **

Dear Professor Hofmann,

Your manuscript titled "Retreaded tires: an overlooked source of microplastics with distinct additive leaching and ecotoxicity" has now been seen by 3 reviewers, and we include their comments at the end of this message. They find your work of interest, but some important points are raised. We are interested in the possibility of publishing your study in Communications Earth & Environment, but would like to consider your responses to these concerns and assess a revised manuscript before we make a final decision on publication.

Additionally, the following points should be addressed in the revised manuscript:

* Clearly present novel insights into the leaching of additives and microplastic from retreaded tires.
* Provide clarify the scope of leaching and the hazard framing.
* Address formulation heterogeneity and implications for representativeness.
* Strengthen the linkage between chemistry and toxicological outcomes
* Clearly discuss the sampling methodology and metadata as asked by the reviewers.

We therefore invite you to revise and resubmit your manuscript, along with a point-by-point response that takes into account the points raised. Please highlight all changes in the manuscript text file.

Please submit your point-by-point responses as a separate file, distinct from your cover letter where you can add responses to the Editors' comments that you do not want to be made available to the reviewers. Word files are preferred. We recommend that any figures, tables or graphs that are included in the response to reviewers are also included in the main article or Supplementary Information.

We are committed to providing a fair and constructive peer-review process. Please don't hesitate to contact us if you wish to discuss the revision in more detail.

Please use the following link to submit your revised manuscript, point-by-point response to the referees' comments (which should be in a separate document to any cover letter), a tracked-changes version of the manuscript (as a PDF file) and the completed checklist:
Link Redacted
** This url links to your confidential home page and associated information about manuscripts you may have submitted or be reviewing for us. If you wish to forward this email to co-authors, please delete the link to your homepage first **

We hope to receive your revised paper within six weeks; please let us know if you aren't able to submit it within this time so that we can discuss how best to proceed. If we don't hear from you, and the revision process takes significantly longer, we may close your file. In this event, we will still be happy to reconsider your paper at a later date, as long as nothing similar has been accepted for publication at Communications Earth & Environment or published elsewhere in the meantime.

Please do not hesitate to contact us if you have any questions or would like to discuss these revisions further. We look

forward to seeing the revised manuscript and thank you for the opportunity to review your work.

Best regards,

Suryasarathi Bose,PhD
Editorial Board Member
Communications Earth & Environment
orcid.org/0000-0001-8043-9192

Somaparna Ghosh, PhD
Associate Editor - Communications Earth & Environment
Consulting Editor - Communications Sustainability

EDITORIAL POLICIES AND FORMATTING

We ask that you ensure your manuscript complies with our editorial policies. Please ensure that the following formatting requirements are met, and any checklist relevant to your research is completed and uploaded as a Related Manuscript file type with the revised article.

For Manuscripts that fall into the following fields:
• Behavioural and social science
• Ecological, evolutionary & environmental sciences
• Life sciences
An updated and completed version of our Reporting Summary must be uploaded with the revised manuscript
You can download the form here:
https://www.nature.com/documents/nr-reporting-summary.pdf

Furthermore, please align your manuscript with our format requirements, which are summarized on the following checklist: Communications Earth & Environment formatting checklist

and also in our style and formatting guide Communications Earth & Environment formatting guide .

*** DATA: Communications Earth & Environment endorses the principles of the Enabling FAIR data project (http://www.copdess.org/enabling-fair-data-project/). We ask authors to make the data that support their conclusions available in permanent, publically accessible data repositories. (Please contact the editor if you are unable to make your data available).

All Communications Earth & Environment manuscripts must include a section titled "Data Availability" at the end of the Methods section or main text (if no Methods). More information on this policy, is available at http://www.nature.com/authors/policies/data/data-availability-statements-data-citations.pdf.

In particular, the Data availability statement should include:
- Unique identifiers (such as DOIs and hyperlinks for datasets in public repositories)
- Accession codes where appropriate
- If applicable, a statement regarding data available with restrictions
- If a dataset has a Digital Object Identifier (DOI) as its unique identifier, we strongly encourage including this in the Reference list and citing the dataset in the Data Availability Statement.

DATA SOURCES: All new data associated with the paper should be placed in a persistent repository where they can be freely and enduringly accessed. We recommend submitting the data to discipline-specific, community-recognized repositories, where possible and a list of recommended repositories is provided at http://www.nature.com/sdata/policies/repositories.

If a community resource is unavailable, data can be submitted to generalist repositories such as figshare or Dryad Digital Repository. Please provide a unique identifier for the data (for example a DOI or a permanent URL) in the data availability statement, if possible. If the repository does not provide identifiers, we encourage authors to supply the search terms that will return the data. For data that have been obtained from publically available sources, please provide a URL and the specific data product name in the data availability statement. Data with a DOI should be further cited in the methods reference section.

Please refer to our data policies at http://www.nature.com/authors/policies/availability.html.

REVIEWER COMMENTS:

Reviewer #1 (Remarks to the Author):

The authors present an interesting study regarding the leaching of additives from retreaded tires. The findings are globally relevant and provide a valuable contribution for the scientific community. It is easy to follow the line of argumentation, and the authors provided comprehensive information on the applied methodology.

I have a few comments and suggestions for further clarification and improvement of the manuscript which I hope the authors find helpful.

Line 40: I agree that leaching of chemicals from TWP is a major problem. However, there are two points that could be clarified or added in this sentence: a) Leaching is not restricted to the additives, but degradation products from additives and degradation products from the rubber may leach from the TWPs as well. b) The TWP as a particle might also cause harm.

Line 87, first word: This seems to be a typo, I guess “RT” is meant instead of “NT”.

Lines 225-228, 235-239, and 279 ff: TWP release rate:
Line 279 ff: The TWPs were generated by cryomilling. This is a valid method to produce samples. However, one question remains: What is the TWP release rate under realistic conditions (use phase)? The release rate might be different for new, used, and retreaded tires. The leaching of chemicals then needs to be related to the total particle release. This also refers to lines 225-228: The environmental risk not only depends on the leaching, but also on the number of TWP that are released on the road.
Similarly, in Lines 235-239: Although details are given in Text S6, I encourage the authors to include some actual numbers on tire wear release of the different tire types (NT, UT, RT) in the main text as well. This is a very important aspect and could be combined with the study results regarding the leaching of chemicals, further improving the relevance of this study. It deserves more attention in the main text, as it could provide a clear projection of actual quantities of released chemicals from tire wear.

Supplementary info, line 191: Especially for the used tires, information on the mileage could provide interesting insights, in addition to the manufacturing date that is given in table S9. In figure 1C and 2B, there is a large deviation between the used tire samples. Can a part of this deviation be explained by differences in the mileage or condition of the specific tire?

Reviewer #2 (Remarks to the Author):

This study investigates tire wear particles (TWPs) originating from retreaded tires, an overlooked yet increasingly important microplastic source. Although retreaded-tire TWPs contain lower total additive mass than new tires, they exhibit significantly higher additive leaching potential, especially for p-phenylenediamines (PPDs) such as IPPD. Three retreaded-tire samples with high IPPD content showed markedly stronger ecotoxicity toward Vibrio fischeri and Chlorella vulgaris. Modeling under the SSP2 scenario projects a multi-hundred-fold global increase in retreaded-tire TWP emissions by 2060. The work highlights substantial environmental risks associated with retreaded tires and underscores the need for regulatory attention and broader ecotoxicological evaluation.

1. The manuscript highlights heterogeneity in retreaded-tire formulations, yet the implications for sample selection, reproducibility, and generalizability could be discussed more explicitly. A dedicated subsection explaining how manufacturing variability affects environmental risk would strengthen the narrative.

2. While IPPD and 4HDPA are identified as key toxicity drivers, the mechanistic explanation (e.g., oxidative stress pathways, membrane disruption) is brief. More integration of chemical behavior with toxicological endpoints would enhance interpretability.

3.The discussion mentions the need for stricter regulation, but specific recommendations, such as harmonized additive disclosure, retreading quality-control standards, or phase-out strategies for high-risk antioxidants, would increase the manuscript’s applied value.

Reviewer #3 (Remarks to the Author):

This manuscript reports a review of the generation of microplastics from the retreaded tires. Generation of microplastics from different polymers has been of the major issue, as it causes global pollution. There have been many research articles on this topic. In this article, the authors have specifically reported the microplastics from the retreaded tires.
The manuscript should have been properly structured and narrated with a suitable comparison with the control sample. Like, what’s the difference in the production of microplastics from the retreaded tire vs the generation of the same by the new tire tread.
1. Will this be called as microplastics, though they are generated from the treaded tyre?
2. The utilization of 6-PPD has been a major concern to the environment. What could be the remedy for the same.?
3. A suitable perspective, precise description and literature review on this topic would have better visibility for the reader

community working in this field!
4. What could be the possible remedy for the reduction of microplastics in the environment!

** Visit Nature Portfolio's author and referees' website at www.nature.com/authors for information about policies, services and author benefits**

Communications Earth & Environment is committed to improving transparency in authorship. As part of our efforts in this direction, we are now requesting that all authors identified as 'corresponding author' create and link their Open Researcher and Contributor Identifier (ORCID) with their account on the Manuscript Tracking System prior to acceptance. ORCID helps the scientific community achieve unambiguous attribution of all scholarly contributions. You can create and link your ORCID from the home page of the Manuscript Tracking System by clicking on 'Modify my Springer Nature account' and following the instructions in the link below. Please also inform all co-authors that they can add their ORCIDs to their accounts and that they must do so prior to acceptance.
https://www.springernature.com/gp/researchers/orcid/orcid-for-nature-research

For more information please visit http://www.springernature.com/orcid

If you experience problems in linking your ORCID, please contact the Platform Support Helpdesk.

Version 1:

Decision Letter:

** Please ensure you delete the link to your author home page in this e-mail if you wish to forward it to your coauthors **

Dear Professor Hofmann,

Your manuscript titled "Retreaded tires: an overlooked source of microplastics with distinct additive leaching and ecotoxicity" has now been seen by our reviewers, whose comments appear below. In light of their advice we are delighted to say that we are happy, in principle, to publish a suitably revised version in Communications Earth & Environment.

We therefore invite you to revise your paper one last time to address the remaining concerns of our reviewers. At the same time we ask that you edit your manuscript to comply with our format requirements and to maximise the accessibility and therefore the impact of your work.

EDITORIAL REQUESTS:

Please review our specific editorial comments and requests regarding your manuscript in the attached "Editorial Requests Table".

*****Please take care to match our formatting and policy requirements. We will check revised manuscript and return manuscripts that do not comply. Such requests will lead to delays. *****

Please outline your response to each request in the right hand column. Please upload the completed table with your manuscript files as a Related Manuscript file.

If you have any questions or concerns about any of our requests, please do not hesitate to contact me.

SUBMISSION INFORMATION:

In order to accept your paper, we require the files listed at the end of the Editorial Requests Table; the list of required files is also available at https://www.nature.com/documents/commsj-file-checklist.pdf .

OPEN ACCESS:

Communications Earth & Environment is a fully open access journal. Articles are made freely accessible on publication. For further information about article processing charges, open access funding, and advice and support from Nature Portfolio, please visit https://www.nature.com/commsenv/open-access

At acceptance, you will be provided with instructions for completing the open access licence agreement on behalf of all authors. This grants us the necessary permissions to publish your paper. Additionally, you will be asked to declare that all

required third party permissions have been obtained, and to provide billing information in order to pay the article-processing charge (APC).

Please use the following link to submit the above items:
Link Redacted
** This url links to your confidential home page and associated information about manuscripts you may have submitted or be reviewing for us. If you wish to forward this email to co-authors, please delete the link to your homepage first **

We hope to hear from you within two weeks; please let us know if you need more time.

Best regards,

Somaparna Ghosh, PhD
Associate Editor,
Communications Earth & Environment
Consulting Editor,
Communications Sustainability

REVIEWERS' COMMENTS:

Reviewer #1 (Remarks to the Author):

All my comments were addressed; I have no further comments. I congratulate the authors to this interesting and relevant contribution.

Reviewer #2 (Remarks to the Author):

The authors have answered all my queries. Hence this may be accepted.

** Visit Nature Portfolio's author and referees' website at www.nature.com/authors for information about policies, services and author benefits**

Open Access This Peer Review File is licensed under a Creative Commons Attribution 4.0 International License, which permits use, sharing, adaptation, distribution and reproduction in any medium or format, as long as you give appropriate

credit to the original author(s) and the source, provide a link to the Creative Commons license, and indicate if changes were made.
In cases where reviewers are anonymous, credit should be given to 'Anonymous Referee' and the source.
The images or other third party material in this Peer Review File are included in the article's Creative Commons license, unless indicated otherwise in a credit line to the material. If material is not included in the article's Creative Commons license and your intended use is not permitted by statutory regulation or exceeds the permitted use, you will need to obtain permission directly from the copyright holder.
To view a copy of this license, visit https://creativecommons.org/licenses/by/4.0/

Manuscript ID: COMMSENV-25-4682

Title: Retreaded tires: an overlooked source of microplastics with distinct additive leaching and ecotoxicity

Author(s): Hao Liu, Tianchi Cao, Yan Lin, Guoliang Shi, Kaiwei Huang, Zhi Cao, Tong Zhang, Thilo Hofmann, and Wei Chen

Below are our responses to the comments from the editor and reviewers. The comments are repeated and then our response is typed. The revised text is shown in blue font.

Response to Reviewers' Comments

Reviewer #1

General Comment	*The authors present an interesting study regarding the leaching of additives from retreaded tires. The findings are globally relevant and provide a valuable contribution for the scientific community. It is easy to follow the line of argumentation, and the authors provided comprehensive information on the applied methodology.*<br><br>*I have a few comments and suggestions for further clarification and improvement of the manuscript which I hope the authors find helpful.*
Response	We sincerely appreciate the reviewer's positive evaluation and constructive suggestions. As recommended, we have carefully revised the manuscript to improve clarity and strengthen the discussion, as detailed in our point-by-point responses below.
Comment	*Line 40: I agree that leaching of chemicals from TWP is a major problem. However, there are two points that could be clarified or added in this sentence: a) Leaching is not restricted to the additives, but degradation products from additives and degradation products from the rubber may leach from the TWPs as well. b) The TWP as a particle might also cause harm.*
Response	We thank the reviewer for this important clarification. We have revised both the Introduction and Discussion to explicitly acknowledge that (i) leaching from TWPs includes not only intentionally added additives but also their transformation products and rubber-derived degradation compounds, and (ii) TWPs may exert ecological impacts as particulate materials independent of chemical release. The revised Introduction (Lines 41–47) now integrates both particle-mediated effects

and chemical release processes in a single framing paragraph. In addition, the Discussion (Lines 276–278) explicitly states that leaching encompasses parent additives and their potentially more toxic transformation products, thereby clarifying the multi-dimensional nature of TWP-associated hazard.

> "TWPs are particulate, polymer-containing materials consisting primarily of natural and synthetic rubber, carbon black, and associated organic and inorganic components[11]. Beyond their physical-mechanical impacts (such as particle ingestion and subsequent impairments to reproduction and survival in freshwater organisms[12, 13]), the major concern regarding TWPs lies in the leaching of chemical additives—such as antioxidants, plasticizers, and ultraviolet stabilizers[14]—along with their transformation products and rubber-derived degradation compounds[15, 16, 17, 18]."

> "The environmental hazard of retreaded TWPs is a multi-dimensional challenge. The leaching of toxic compounds encompasses not only parent additives (e.g., PPDs) but also their transformation products (e.g., quinones), which are often more toxic."

Comment *Line 87, first word: This seems to be a typo, I guess "RT" is meant instead of "NT".*

Response We thank the reviewer for catching this. It has been corrected (Line 96-97).

> "…TWPs generated from retreaded tires (referred to as "RT" hereafter), …"

Comment *Lines 225-228, 235-239, and 279 ff: TWP release rate: Line 279 ff: The TWPs were generated by cryomilling. This is a valid method to produce samples. However, one question remains: What is the TWP release rate under realistic conditions (use phase)? The release rate might be different for new, used, and retreaded tires. The leaching of chemicals then needs to be related to the total particle release. This also refers to lines 225-228: The environmental risk not only depends on the leaching, but also on the number of TWP that are released on the road, Similarly, in Lines 235-239: Although details are given in Text S6, I encourage the authors to include some actual numbers on tire wear release of the different tire types (NT, UT, RT) in the main text as well. This is a very important aspect and could be combined with the study results regarding the leaching of chemicals, further improving the relevance of this study. It deserves more attention in the main text, as it could provide a clear projection of actual quantities of released chemicals from tire wear.*

Response

We appreciate the reviewer's insightful comment. We agree that the environmental risk associated with retreaded-tire TWPs depends not only on additive leaching and toxicity per unit mass, but also on the magnitude of particle emissions during the use phase.

To evaluate whether differential emission rates could confound our conclusions, we conducted a systematic literature review (Web of Science and Google Scholar) focusing on studies that directly compare TWP generation from retreaded and new tires. This search revealed a clear data gap: only one peer-reviewed study (Wang et al., *Tribology Letters*, 2025) has experimentally compared wear emissions from new tire rubber and rubber incorporating reclaimed material under controlled tribological conditions. That study reported modest and load-dependent differences, with reclaimed-rubber formulations emitting 12–19% more particles in specific size fractions under heavy-load conditions, but similar emissions under light-load scenarios; conversely, new tire rubber showed slightly higher emissions in certain size classes when normalized to worn mass. Taken together, these counterbalancing effects indicate that available evidence does not support systematically higher wear emissions from retreaded tires under typical operating conditions.

We have revised the Discussion (Lines 278–284) to explicitly reflect this state of knowledge and to clarify that, given the current evidence, the distinct chemical composition and leaching behaviour of retreaded-tire TWPs—rather than demonstrably higher emission rates—represent the primary driver of the differentiated environmental risk identified in our study.

> "Beyond chemical leaching, the total environmental burden is dictated by the release rate of the particles themselves. Currently, empirical comparisons of emission rates remain scarce; only one peer-reviewed study has directly compared TWP generation from new tire rubber vs. rubber incorporating reclaimed material under controlled laboratory tribological conditions[52]. While these preliminary data suggest that, under typical scenarios, emission rates from retreaded tires are comparable in magnitude with those of conventional tires,..."

We have further clarified the selection and justification of TWP release rates used in the modelling framework. Current field measurements and emission inventories for conventional (non-retreaded) tires consistently indicate that mass-based TWP generation under realistic driving conditions is primarily governed by vehicle- and use-related factors—such as vehicle mass (axle load), driving behavior (speed, acceleration, braking), and road surface properties—rather than tire type alone (Grigoratos et al., *EUR Report*, 2014; Fussell et al., *Environ. Sci. Technol*., 2022). Consistent with this established evidence base, contemporary emission-inventory approaches parameterize TWP release rates according to vehicle category and

mass rather than tire manufacturing route. Following this framework, we applied vehicle-archetype-specific TWP emission factors in our analysis, distinguishing four vehicle size classes and six powertrain types (ICEG, ICED, HEV, PHEV, BEV, and FCV). The revised manuscript and Supplementary Information (Text S6) now explicitly describe the empirical basis and parameterization strategy underlying these release-rate assumptions.

> "The model defines 48 vehicle archetypes, distinguished by six powertrain types (ICEG, ICED, HEV, PHEV, BEV, and FCV), four size categories (microcars, passenger cars, minivans/SUVs, and light trucks), and two lightweighting levels (conventional and lightweighted). Based on available field measurements and emission inventories, mass-based tire wear particle (TWP) generation under realistic driving conditions is primarily governed by vehicle- and use-related factors, including vehicle mass, driving behavior (speed, acceleration, braking), and road surface properties, rather than tire type alone. Accordingly, the same TWP emission factor was applied to NT, UT, and RT within the same vehicle archetype, while distinct emission factors were assigned across different vehicle categories (Table S14). In the absence of consistent empirical evidence demonstrating systematic differences in use-phase emission rates between new and retreaded tires, this assumption follows current emission-inventory practice and reflects the present state of knowledge."

> "Previous studies have reported a close association between TWP emissions and vehicle mass. Early experimental work suggested an approximately linear dependence of TWP emissions on vehicle load.[15] Beddows and Harrison (2021) further identified vehicle mass as a key determinant when comparing TWP emissions across powertrains (for example, BEV vs. ICE passenger vehicles),[16] and national-scale emissions inventories likewise use vehicle mass to scale TWP emission factors across vehicle fleets.[17] Accordingly, a mass-based parameterization was used to represent differences in TWP emission factors among vehicle archetypes. Given the limited availability of mass-resolved TWP emission-factor data, vehicle-specific TWP emission factors from an independent real-world measurement study[18] were used as reference values for the scaling and paired with mass data compiled from the literature[8] to assign emission factors to the remaining archetypes. The measurement study reports total TWP emission factors for ICEG passenger cars and HEV passenger cars. These categories were mapped to the corresponding passenger-vehicle archetypes (ICEG/HEV, passenger car, non-lightweight), reflecting the baseline (non-lightweighted) vehicle specification in that study, and used to anchor the mass-scaling applied to the remaining archetypes. This yielded the following form:

$$EF_i = 0.0688V_i - 24.583 \quad (S9)$$

where V_i is vehicle mass of archtype i. This parameterization was then used to derive archetype-specific EF_i values, as summarized in Table S14."

Table S14 TWP emission factor for different vehicle categories.

**Archetype**	**TWP emission factor (mg/vehicle/km)**
ICEG, microcar, lightweight	16.53
ICEG, microcar, non-lightweight	25.64
ICEG, passenger car, lightweight	52.95
ICEG, passenger car, non-lightweight	53.00
ICEG, minivan/SUVs, lightweight	72.07
ICEG, minivan/SUVs, non-lightweight	93.27
ICEG, light truck, lightweight	97.19
ICEG, light truck, non-lightweight	115.63
ICED, microcar, lightweight	17.22
ICED, microcar, non-lightweight	26.50
ICED, passenger car, lightweight	54.06
ICED, passenger car, non-lightweight	65.96
ICED, minivan/SUVs, lightweight	73.33
ICED, minivan/SUVs, non-lightweight	94.83
ICED, light truck, lightweight	99.12
ICED, light truck, non-lightweight	117.90
BEV, microcar, lightweight	30.89
BEV, microcar, non-lightweight	47.23
BEV, passenger car, lightweight	74.35
BEV, passenger car, non-lightweight	93.27
BEV, minivan/SUVs, lightweight	92.13
BEV, minivan/SUVs, non-lightweight	125.90
BEV, light truck, lightweight	126.23
BEV, light truck, non-lightweight	155.12
HEV, microcar, lightweight	19.12
HEV, microcar, non-lightweight	30.75
HEV, passenger car, lightweight	57.36
HEV, passenger car, non-lightweight	72.00
HEV, minivan/SUVs, lightweight	75.44
HEV, minivan/SUVs, non-lightweight	101.74

HEV, light truck, lightweight	102.28
HEV, light truck, non-lightweight	128.02
PHEV, microcar, lightweight	19.54
PHEV, microcar, non-lightweight	33.09
PHEV, passenger car, lightweight	58.25
PHEV, passenger car, non-lightweight	74.97
PHEV, minivan/SUVs, lightweight	75.08
PHEV, minivan/SUVs, non-lightweight	105.26
PHEV, light truck, lightweight	102.28
PHEV, light truck, non-lightweight	128.02
FCV, microcar, lightweight	18.26
FCV, microcar, non-lightweight	31.64
FCV, passenger car, lightweight	56.60
FCV, passenger car, non-lightweight	73.32
FCV, minivan/SUVs, lightweight	76.04
FCV, minivan/SUVs, non-lightweight	107.18
FCV, light truck, lightweight	101.46
FCV, light truck, non-lightweight	127.47

We recognize that quantifying TWP release under real-world conditions remains a complex and evolving research area. Emission processes are influenced by the interplay of vehicle dynamics, tire formulation heterogeneity, road surface properties and climatic conditions, and systematic comparisons between retreaded and new tires under realistic driving scenarios remain limited. We have revised the manuscript (Lines 284–290) to explicitly acknowledge this limitation and to clarify that improved empirical constraints on use-phase emissions would further refine model projections.

> "…it is important to acknowledge that real-world TWP release is governed by the complex interplay of vehicle load, driving behavior, road conditions, and tire formulation. In the absence of systematic comparative field data for retreaded vs. new tires, current emission-factor parameterizations necessarily rely on vehicle-mass scaling approaches. Consequently, future investigations should integrate realistic driving experiments with high-resolution tire characterization to better constrain use-phase emissions of retreaded vs. new and worn tires."

Comment *Supplementary info, line 191: Especially for the used tires, information on the mileage could provide interesting insights, in addition to the manufacturing date*

that is given in table S9. In figure 1C and 2B, there is a large deviation between the used tire samples. Can a part of this deviation be explained by differences in the mileage or condition of the specific tire?

Response We thank the reviewer for this important and insightful comment. We agree that differences in mileage and service history may contribute to the variability observed among UT samples in Figures 1C and 2B. Although detailed mileage records were not available for the used tires (as such information is typically not retained by retailers), we evaluated indirect proxies of service history, including tread-depth reduction and surface oxidation (O/C atomic ratio), which are established indicators of cumulative wear and chemical aging (Giechaskiel et al., *Vehicles*, 2025; Zhou et al., *Industrial Crops and Products*, 2025). These additional analyses, now presented in Fig. S1, indicate that neither total additive mass nor leachability exhibited clear correlations with these service-history proxies. The manuscript has been revised accordingly (Lines 100–105; 141–145) to clarify that the observed variability among UT samples cannot be simply attributed to mileage differences.

> "Between 20 and 27 of the 40 target additives were detected in the 9 RT samples; for the 7 NT samples, 23 to 30 additives were detected, whereas for the 11 UT samples, the number of detected additives ranged from 15 to 26. (The large variability among the UT samples cannot simply be attributed to their service history, as the mass of additives displayed no clear correlations with tread depth reduction or surface O/C ratio, used as a proxy for mileage (Fig. S1A,B)[35, 36].)."

> "The PCA results revealed that the chemical distribution and concentration patterns in the leachates varied among all three groups of TWPs (Fig. 2A), even though the compositional profiles of the additives in the TWPs were similar between RTs and NTs (Fig. 1B). (Again, for the UTs no clear correlations were observed between leachable additives and service history; Fig. S1C,D.)."

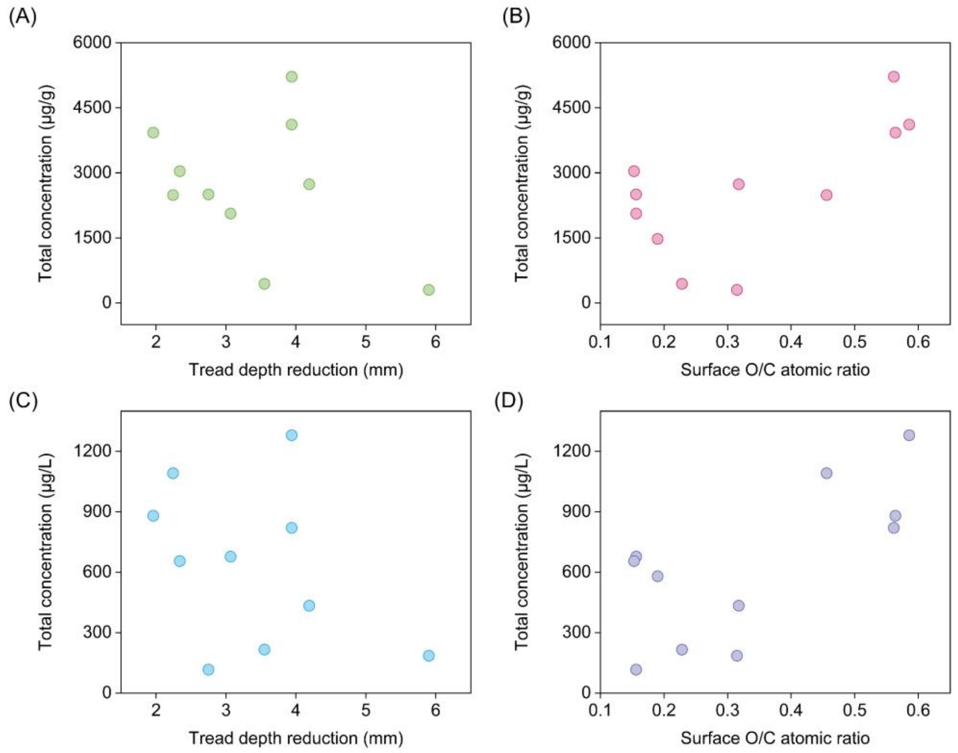

Fig. S1. The total concentration of chemical additives in TWPs (A and B) and in TWP leachates (C and D), as a function of tread depth reduction (i.e., thickness of tread removed during service) and surface oxidation, expressed as the O/C atomic ratio measured by XPS.

Reviewer #2

General Comment	*This study investigates tire wear particles (TWPs) originating from retreaded tires, an overlooked yet increasingly important microplastic source. Although retreaded-tire TWPs contain lower total additive mass than new tires, they exhibit significantly higher additive leaching potential, especially for p-phenylenediamines (PPDs) such as IPPD. Three retreaded-tire samples with high IPPD content showed markedly stronger ecotoxicity toward Vibrio fischeri and Chlorella vulgaris. Modeling under the SSP2 scenario projects a multi-hundred-fold global increase in retreaded-tire TWP emissions by 2060. The work highlights substantial environmental risks associated with retreaded tires and underscores the need for regulatory attention and broader ecotoxicological evaluation.*

Response We sincerely appreciate the reviewer's positive evaluation of our work and the constructive comments, which have helped us further strengthen the manuscript.

Comment *1. The manuscript highlights heterogeneity in retreaded-tire formulations, yet the implications for sample selection, reproducibility, and generalizability could be discussed more explicitly. A dedicated subsection explaining how manufacturing variability affects environmental risk would strengthen the narrative.*

Response We sincerely thank the reviewer for this constructive and insightful suggestion. We agree that the inherent heterogeneity of retreaded-tire formulations is a critical factor influencing environmental risk profiles, reproducibility, and the generalizability of experimental findings. In response, we have added a dedicated subsection in the Discussion (3rd paragraph, Lines 258–275) to explicitly address how manufacturing variability affects environmental risk assessment.

> "The inherent heterogeneity in retreaded-tire formulations presents a significant challenge for environmental risk assessment and study reproducibility. Unlike new tires manufactured by major producers under standardized production processes, retreaded tires originate from a comparatively fragmented sector, involving diverse tread compounds and retreading routes (e.g., precure vs. mold-cure), with processing conditions varying across facilities and regions[30, 33, 51]. This variability likely contributes to the pronounced differences in additive leachability observed among retreaded TWPs. Moreover, the discrepancy between total additive mass and leaching potential suggests that retreading processes may modify the rubber matrix structure (e.g., porosity or bonding characteristics), thereby influencing release dynamics. While our SSP2 modelling projects a substantial expansion of retreaded-tire TWP emissions, the associated environmental impact will depend not only on emission magnitude but also on the chemical profile of future retreads. The elevated toxicity observed in samples with high IPPD content indicates that formulation heterogeneity may generate localized ecological risk hotspots. Although our sampling strategy aimed to capture realistic formulation diversity across multiple sources, broader characterization across global markets will be required to fully constrain this variability. These findings suggest that risk assessment frameworks should account for formulation-dependent leaching behaviour, rather than relying solely on bulk additive inventories."

Comment *2. While IPPD and 4HDPA are identified as key toxicity drivers, the mechanistic explanation (e.g., oxidative stress pathways, membrane disruption) is brief. More*

integration of chemical behavior with toxicological endpoints would enhance interpretability.

Response

We thank the reviewer for this insightful comment. We agree that a clearer integration of chemical behavior and toxicological endpoints strengthens interpretability. In addition to expanding the mechanistic discussion, we now more explicitly link additive composition to biological response using our multivariate analyses. Specifically, redundancy analysis and random forest classification identify IPPD and 4HDPA as major contributors to the observed inhibition of *Vibrio fischeri* luminescence and *Chlorella vulgaris* growth, accounting together for 33.1% of overall toxicity—comparable to 6PPD (33.8%).

To interpret these statistical associations mechanistically, we integrated available evidence from structurally related PPD antioxidants (e.g., 6PPD and 6PPD-quinone) with the physicochemical characteristics of IPPD and 4HDPA (Lines 207-216). While direct mechanistic studies on IPPD and 4HDPA in microalgae remain limited, studies on the broader PPD class consistently demonstrate (i) oxidative stress induction via redox cycling and reactive oxygen species (ROS) formation (Liu et al., *Sci. Total Environ*., 2024); (ii) membrane perturbation due to hydrophobic aromatic structure, affecting permeability and photosynthetic/mitochondrial function (Yan et al., *Sci. Total Environ.*, 2024); and (iii) metabolic interference impacting energy metabolism and nutrient assimilation (Chen et al., *J. Hazard. Mater*., 2024). Given their shared structural and redox characteristics, IPPD and 4HDPA likely act through analogous pathways.

"RFC further indicated that IPPD and 4HDPA made a combined contribution of 33.1% to the overall toxicity, on par with that of 6PPD (33.8%) (Fig. 3D), indicating a strong compound-level association between PPD-type additives and algal growth inhibition. Although the mechanisms controlling the toxicity of IPPD and its transformation product 4HDPA to microalgae have not been directly investigated, studies on structurally related p-phenylenediamine antioxidants, particularly 6PPD and 6PPD-quinone, consistently show that this chemical class induces oxidative stress, disrupts membrane integrity, and interferes with photosynthesis and nutrient metabolism in microalgae[44, 45, 46]. Given these shared aromatic structural and redox characteristics among PPD derivatives, IPPD and 4HDPA likely inhibited algal growth through similar mechanisms."

Comment

3. The discussion mentions the need for stricter regulation, but specific recommendations, such as harmonized additive disclosure, retreading quality-

control standards, or phase-out strategies for high-risk antioxidants, would increase the manuscript's applied value.

Response We thank the reviewer for this constructive suggestion. We agree that articulating clearer regulatory implications strengthens the applied relevance of the study. Accordingly, we have revised the Discussion (5th paragraph, Lines 295–309) to specify potential intervention strategies grounded in our findings.

"Addressing the risks associated with PPDs and their transformation products will likely require coordinated regulatory and technical approaches. In the near term, downstream stormwater control measures—such as bioretention systems and other green infrastructure—can help intercept roadway-derived chemical loads. Longer-term mitigation may involve upstream measures, including harmonized disclosure of tire and retread formulations, strengthened quality-control standards in retreading practices to reduce formulation-driven variability, and risk-based evaluation or substitution of high-concern antioxidants. Beyond additive-specific concerns, reducing the environmental burden of TWPs will require strategies that limit particle generation and environmental release. Potential approaches include improving material durability and tread design to reduce abrasion-derived emissions, optimizing vehicle operation and maintenance practices, and enhancing interception of tire-derived particles in stormwater and urban runoff systems. Integrating such source-control and interception strategies with additive risk management may provide a more comprehensive framework for mitigating tire-derived microplastic pollution across jurisdictions."

Reviewer #3

General Comment *This manuscript reports a review of the generation of microplastics from the retreaded tires. Generation of microplastics from different polymers has been of the major issue, as it causes global pollution. There have been many research articles on this topic. In this article, the authors have specifically reported the microplastics from the retreaded tires.*

The manuscript should have been properly structured and narrated with a suitable comparison with the control sample. Like, what's the difference in the production of microplastics from the retreaded tire vs the generation of the same by the new tire tread.

Response We thank the reviewer for this constructive comment. We would like to clarify that this study presents an experimental and comparative investigation rather than a review, systematically benchmarking retreaded tires against new and used tires

to define their distinct chemical, toxicological, and projected environmental footprint. We agree that a clearly structured comparison between retreaded and conventional tires is essential, and we have revised the manuscript accordingly to strengthen this benchmarking framework.

1) Clarification of benchmarking: We have revised the Introduction (Lines 57–60, 73–77) to explicitly state that new and used tires were employed as experimental reference controls to quantify how retreaded tires deviate from standard market products in terms of chemical composition, additive leaching behavior, toxicological potency, and structural characteristics relevant to TWP generation.

> "Retreads are structurally distinct from both new and worn tires – while the latter two feature an integral tread-to-casing structure (i.e., the tread and casing are manufactured as a single homogeneous unit), retreaded tires may possess a vulcanized interface between the old casing and the new tread[29,30]."

> "The primary aim of this study is to investigate the distinct environmental risks of TWPs from retreaded tires, including the leaching of toxic additives and transformation products, by systematically comparing their chemical compositions, additive leaching behavior, and leachate ecotoxicity against those from new and used tires, which serve as critical reference controls for benchmarking."

2) Addressing differences in particle generation: We agree that the total environmental burden of TWPs is determined by both toxicity per unit mass and particle emission rates. While the experimental component of our study focused on chemical composition and leaching behavior, we have expanded the Discussion (4th paragraph, Lines 276–284) to address current knowledge regarding mechanical TWP generation. Empirical comparisons of emission rates between retreaded and new tires remain scarce; to our knowledge, only one peer-reviewed study has directly compared TWP generation from new tire rubber and rubber incorporating reclaimed material under controlled tribological conditions (Wang et al., *Tribology Letters*, 2025). These data suggest broadly comparable wear rates under typical scenarios. We further note that the structural characteristics of retreaded tires—such as the vulcanized interface between casing and tread—may influence fragmentation behavior under real-world conditions, although systematic field validation remains limited.

> "The environmental hazard of retreaded TWPs is a multi-dimensional challenge. The leaching of toxic compounds encompasses not only parent additives (e.g., PPDs) but also their transformation products (e.g., quinones), which are often more toxic. Beyond chemical leaching, the total environmental burden is

dictated by the release rate of the particles themselves. Currently, empirical comparisons of emission rates remain scarce; only one peer-reviewed study has directly compared TWP generation from new tire rubber vs. rubber incorporating reclaimed material under controlled laboratory tribological conditions[52]. While these preliminary data suggest that, under typical scenarios, emission rates from retreaded tires are comparable in magnitude with those of conventional tires,….”

3) Identifying research gaps: We have emphasized that the potential difference in particle emission rates between retreaded and new tires remains a critical research gap (Discussion, Lines 284–290), which our study complements by providing a detailed chemical–toxicological characterization of retreaded-tire TWPs relative to new and used tires.

“…it is important to acknowledge that real-world TWP release is governed by the complex interplay of vehicle load, driving behavior, road conditions, and tire formulation. In the absence of systematic comparative field data for retreaded vs. new tires, current emission-factor parameterizations necessarily rely on vehicle-mass scaling approaches. Consequently, future investigations should integrate realistic driving experiments with high-resolution tire characterization to better constrain use-phase emissions of retreaded vs. new and worn tires.”

Comment *1. Will this be called as microplastics, though they are generated from the treaded tyre?*

Response We thank the reviewer for this conceptual clarification. Tire wear particles (TWPs) are commonly classified as a major category of microplastic pollution in the literature (Goßmann et al., *Nature Communications*, 2023; Evangeliou et al., *Nature Communications*, 2020). TWPs meet the widely accepted operational criteria for microplastics: they are solid particulate materials containing substantial fractions of synthetic polymers (e.g., styrene-butadiene rubber and polybutadiene), are typically generated in the micrometer size range through mechanical abrasion, and persist in the environment while exhibiting ecological behaviors similar to other microplastics, including ingestion and chemical leaching. We have revised the Introduction (Lines 36–43) to explicitly define TWPs and clarify their classification within the microplastic framework to avoid ambiguity.

“Tire wear particles (TWPs), a major contributor of microplastic pollution in the environment[1, 2, 3], have attracted increasing attention due to their widespread occurrence and adverse ecological impact[4, 5, 6, 7]. It is estimated that each year approximately 1.4 million metric tons of TWPs are generated globally, primarily

from the mechanical abrasion of tire treads on road surfaces[8, 9]. These TWPs are readily transported to soil, aquatic systems, and air, via road runoff and atmospheric deposition[10]. TWPs are particulate, polymer-containing materials consisting primarily of natural and synthetic rubber, carbon black, and associated organic and inorganic components[11]."

Comment *2. The utilization of 6-PPD has been a major concern to the environment. What could be the remedy for the same.?*

Response We thank the reviewer for this important question regarding potential remedies for 6PPD and related PPD antioxidants. In response, we have revised the Discussion (5th paragraph, Lines 295–302) to provide a structured overview of both downstream mitigation measures and upstream risk-reduction strategies informed by our findings.

"Addressing the risks associated with PPDs and their transformation products will likely require coordinated regulatory and technical approaches. In the near term, downstream stormwater control measures—such as bioretention systems and other green infrastructure—can help intercept roadway-derived chemical loads. Longer-term mitigation may involve upstream measures, including harmonized disclosure of tire and retread formulations, strengthened quality-control standards in retreading practices to reduce formulation-driven variability, and risk-based evaluation or substitution of high-concern antioxidants."

Comment 3. A suitable perspective, precise description and literature review on this topic would have better visibility for the reader community working in this field!

Response We thank the reviewer for this suggestion. To improve clarity, positioning, and relevance for the broader research community, we have revised the Introduction (1st paragraph, Lines 41-47; 2nd paragraph, Lines 57-60) and Discussion (5th paragraph, Lines 291–302) to provide a more explicit conceptual framing of retreaded tires within the broader microplastic and tire-additive literature. Specifically, we have expanded the contextualization of TWPs as a major microplastic source, clarified how retreaded tires differ structurally and chemically from new and used tires, and integrated recent global assessments and regulatory perspectives on tire additives. These revisions strengthen the manuscript's narrative structure and situate our experimental findings within the wider discourse on microplastic pollution and additive management.

"TWPs are particulate, polymer-containing materials consisting primarily of natural and synthetic rubber, carbon black, and associated organic and inorganic components[11]. Beyond their physical-mechanical impacts (such as particle ingestion and subsequent impairments to reproduction and survival in freshwater organisms[12, 13]), the major concern regarding TWPs lies in the leaching of chemical additives—such as antioxidants, plasticizers, and ultraviolet stabilizers[14]—along with their transformation products and rubber-derived degradation compounds[15, 16, 17, 18]."

"Retreads are structurally distinct from both new and worn tires – while the latter two feature an integral tread-to-casing structure (i.e., the tread and casing are manufactured as a single homogeneous unit), retreaded tires may possess a vulcanized interface between the old casing and the new tread[29, 30]."

"Recent global assessments emphasize the importance of transparency in tire chemical composition and international coordination in managing tire additives[15]. Although IPPD has been classified as a Category 1 aquatic acute/chronic toxicant in European regulatory schemes since 1967 [53], and has been largely replaced by 6PPD in some regions [54,55], our results indicate that this high-risk compound remains present in parts of the global tire inventory. Addressing the risks associated with PPDs and their transformation products will likely require coordinated regulatory and technical approaches. In the near term, downstream stormwater control measures—such as bioretention systems and other green infrastructure—can help intercept roadway-derived chemical loads. Longer-term mitigation may involve upstream measures, including harmonized disclosure of tire and retread formulations, strengthened quality-control standards in retreading practices to reduce formulation-driven variability, and risk-based evaluation or substitution of high-concern antioxidants."

Comment 4. What could be the possible remedy for the reduction of microplastics in the environment!

Response We thank the reviewer for this important question regarding broader mitigation of microplastic pollution. We agree that reducing the environmental burden of tire-derived microplastics requires interventions that extend beyond additive management and address both particle generation and environmental transport pathways. Accordingly, we have expanded the Discussion (Lines 302–309) to articulate mitigation strategies at three levels: (i) source reduction through improved material durability and abrasion-resistant tire design; (ii) operational measures to reduce wear-associated emissions; and (iii) interception of particles

in roadway runoff and urban drainage systems. These revisions place the findings of our study within a broader microplastic management framework.

"Beyond additive-specific concerns, reducing the environmental burden of TWPs will require strategies that limit particle generation and environmental release. Potential approaches include improving material durability and tread design to reduce abrasion-derived emissions, optimizing vehicle operation and maintenance practices, and enhancing interception of tire-derived particles in stormwater and urban runoff systems. Integrating such source-control and interception strategies with additive risk management may provide a more comprehensive framework for mitigating tire-derived microplastic pollution across jurisdictions."
